# Global thyroid cancer incidence trend and age-period-cohort model analysis based on Global Burden of Disease Study from 1990 to 2019

**DOI:** 10.3389/fendo.2023.1133098

**Published:** 2023-04-12

**Authors:** Le Xu, Zhe Xu Cao, Xin Weng, Can Fei Wang

**Affiliations:** ^1^ Department of Head and Neck Surgery, Ren Ji Hospital, Shanghai Jiao Tong University School of Medicine, Shanghai, China; ^2^ Department of Thyroid Surgery, The Second Xiangya Hospital, Central South University, Changsha, Hunan, China; ^3^ Department of Pathology, The First Affiliated Hospital, Nanhua University, Hengyang, Hunan, China; ^4^ Department of Hematology, The Second Xiangya Hospital, Central South University, Changsha, Hunan, China

**Keywords:** Global Burden of Disease Study, age-period-cohort model, thyroid cancer, incidence, overdiagnosis

## Abstract

**Background:**

In view of the rapid increase in the incidence of thyroid cancer (TC) and the spread of overdiagnosis around the world, the quantitative evaluation of the effect of age, period and birth cohort on the incidence of TC, and the analysis of the role of different factors in the incidence trend can provide scientific basis and data support for the national health departments to formulate reasonable prevention and treatment policies.

**Methods:**

The study collated the global burden disease study data of TC incidence from 1990 to 2019, and used APC model to analyze the contribution of age, period and birth cohort to the incidence trend of TC.

**Results:**

There was an obvious unfavorable upward trend in terms of age and cohort effect all over the world. Since 2007, the growth rate of risk slowed down and the risk in female even decreased since 2012, which mainly contributed to the developed countries. In all SDI countries, 2002 is the dividing point of risk between male and female. In 2019, The global age-standardized incidence rate (ASIR) of TC in the 5 SDI countries all showed a significant upward trend, with the largest upward trend in the middle SDI countries.

**Conclusion:**

The trend of rapid increase in the incidence of TC has begun to slow down, but the global incidence of TC has obvious gender and regional/national heterogeneity. Policy makers should tailor specific local strategies to the risk factors of each country to further reduce the burden of TC.

## Introduction

With the rapid increase of incidence, thyroid cancer (TC) has become one of the common research hotspots in head and neck surgery, endocrinology and oncology (1). Although TC accounts for only 2% of all malignant tumors, and with favorable prognosis, it may still seriously affect the quality of life of the patients (2–4). In view of the continuous increasing of TC, monitoring its incidence and studying its trend are conducive to the development of tumor prevention and treatment and provide decision-making basis for health administrative departments. Meanwhile, it can also provide valuable clues for further epidemiological study of the etiology of TC (5).

The Global Burden of Disease, Injuries, and Risk Factors Study (GBD) is a research project led by the Institute for Health Metrics and Evaluation (IHME) which covers 204 countries and territories around the world, and aims to quantitatively evaluate the disease burden and health status of people in various countries (6). Previous studies used outdated data to explore the incidence of TC, while GBD 2019 provided the latest population-based cancer registration data, which estimated the burden of 285 diseases and disabilities using a uniform and standardized approach. This study will compare the data of different countries/regions and different periods through GBD 2019 in order to provide data support for the rational allocation of medical resources and the establishment of prevention and screening system (7).

It is widely known that age, period and birth cohort are the three internal factors that play an important role in malignant tumors. However, there is an absolute linear relationship between these three factors so that the traditional quantitative description methods can not address the issue of their interaction. The emergence of the Age-period-cohort (APC) model and its continuous improvement in methodology provide a solution to this problem. APC model is a statistical method based on Poisson distribution, which can estimate the risk of disease incidence or death under the condition of adjusting and controlling age, period and cohort at the same time, and makes the incidence trend of cancer more clearly (8).

Age effect refers to the change of disease risk with age, indicating the effect of population structural changes on the incidence of TC. Period effect can be understood as the change of disease risk with the time in the same age group, which can be attributed to the implementation of public health care policy, medical technology innovation and even the change of disease classification (9). Cohort effect means that the long-term trend of disease incidence in people born in different years is affected by different historical, social and environmental conditions. Therefore, when studying the epidemic characteristics and influencing factors of cancer morbidity or incidence, APC model is more widely used (10, 11).

Therefore, our study used comprehensive data of global sources from the GBD research to analyze trends in the incidence of TC at the global, regional and national levels between 1990 and 2019, and on this basis, to estimate the impact of age, period and cohort effect on cancer incidence, which provides an important reference for public health strategies to further reduce the burden of TC.

## Materials and methods

### Data acquisition

The data of this study came from the Global Health Data Exchange Database (GHDx), which was established by the GBD research project and published on the official website (http://ghdx.healthdata.org/) of the University of Washington, USA. The global incidence count and rate of TC from 1990 to 2019 were obtained from this database. GBD Estimate included cause of death or injury, etiology, risk factor, impairment, etc. and in this study, cause of death or injury was selected; Measure included deaths, disability adjusted life year, years of life lost due to premature mortality, years lived with disability, prevalence, incidence and maternal mortality ratio, and in this study incidence was selected; Metric was the unit of measurement, including the number, rate and percentage; Cause was thyroid cancer; Location was a division of regions, in addition to choosing a specific country, it also included supranational levels according to different socio-demographic indices (SDI, that is, the geometric average of the total fertility rate of people under 25, the average education level of people aged 15 and over, and the lagging income per capita), or geographical or custom areas; Age was set as a group every 5 years old, and the population was divided into 19 age groups: 5~9, 10~14, …, and over 95 years old; Sex included all gender, female and male; Year required that each year from 1990 to 2019 be checked.

### Data processing and establishment of APC model

This study used the online tool APC Web Tool (http://analysistools.nci.nih.gov/apc/) provided by the National Cancer Institute for APC analysis. The web tool was supported by built-in estimable function algorithm and corresponding Wald test (12). The parameters involved included: longitudinal age curve, which is the incidence curve of a specific age group fitted in a specific cohort after adjusted period effect and can be regarded as the age effect; period rate ratio, that is, after adjusting the cohort effect, the incidence ratio of a specific period relative to the reference period in a specific age group. Cohort rate ratio, that is, the incidence ratio of a specific cohort to the reference cohort in a specific age group after adjusting the period effect. The diagnosis period was set as a group every 5 years, and seven time points were taken from 1990 to 2019 as observation points: 1990, 1995, 2000, 2005, 2010, 2015 and 2020. The birth cohort was calculated as: cohort = period-age.

The GBD world population age standard was used to calculate age-standardized rates presented throughout GBD (13). The standard population age structure was generated by taking the non-weighted mean of the 2010 to 2035 age-specific proportional distributions for national locations reported by the UN Population Division World Population Prospects 2012 revision. In GBD 2017 and 2019, we used the non-weighted mean of the GBD year’s age-specific proportional distributions for national locations with populations greater than 5 million in the GBD year to update the world population age standard. The final values used for the age standard can be found in **Supplementary Table 1**. In this study, Wald test was used to test the hypothesis of the parameters, and the bilateral Wald test p < 0.05 was considered that the difference is statistically significant, and the p values for Wald test were shown in **Supplementary Table 2**.

## Results

### The age trend of the incidence of TC

**Figure 1:**
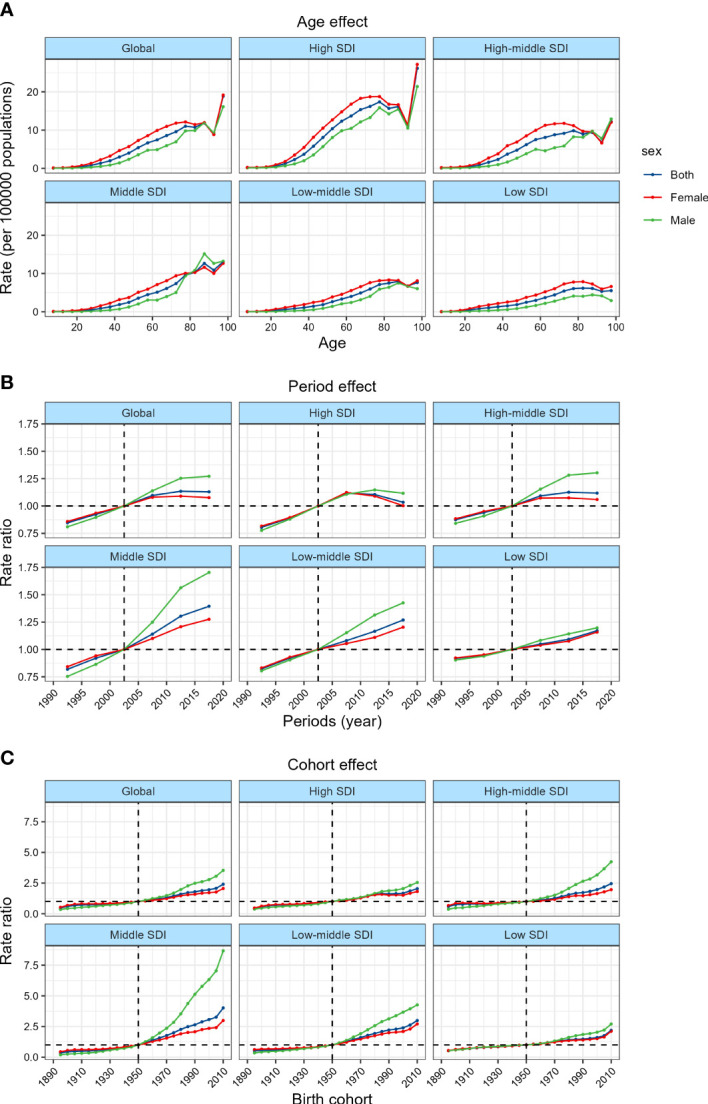
Age, period and cohort effects on TC incidence by SDI quintiles. **(A)** Age effects are shown by the fitted longitudinal age curves of incidence (per 100,000 person-years) adjusted for period deviations. **(B)** Period effects are shown by the relative risk of incidence (incidence rate ratio) and computed as the ratio of age-specific rates from 1990~1994 to 2015~2019 (2000~2005 as the referent period). **(C)** Cohort effects are shown by the relative risk of incidence and computed as the ratio of age-specific rates from the 1895 cohort to the 2010 cohort, with the referent cohort set at 1950.

**Figure 1A** showed the trend of the global incidence of TC with age during the observation period. The age trend of TC can be divided into 4 stages. In those aged before 78, the incidence increased steadily with the increase of age. In those aged 78~84 years old, the incidence decreased slightly and then increased, and in those aged 84~91 years old, the incidence decreased significantly with the increase of age. The incidence rate increased rapidly again with age and reached a peak of nearly 20/100,000 when the age was over 90 years old. In terms of gender differences, the incidence rate of female before 84 years old was always higher than that of male, especially between 60~64 and 70~74 years old, and the maximum difference between the two genders reached 5/100,000. Among different SDI countries, the incidence of TC showed a similar age trend, but the incidence in high SDI countries was significantly higher than that in middle or low SDI countries, and the largest gender difference was shown in high-middle SDI countries. The incidence of female in low SDI countries was higher than that of male in all age groups. Interestingly, the incidence of male in middle SDI countries was higher than that of female over 80 years old.

### The period trend of the incidence of TC

The global TC incidence risk which changes with the period was shown in **Figure 1B**. From 1990 to 2009, the risk of TC increased rapidly with time. From 2010 to 2014, the growth rate of risk slowed down. From 2014 to 2019, the increase in the risk of TC further slowed down, and the risk in female even decreased. Since 2002, the risk in female has changed from higher to lower than that in male, and the risk ratio difference has a further widening trend. Among different SDI countries, the risk of TC increased rapidly at first and then slowed down or even decreased in high-SDI and high-middle SDI countries, but the risk in middle and lower SDI countries showed an upward trend in the whole period. It is worth noting that in all SDI countries, 2000~2004 was the dividing point of risk between male and female.

### The cohort trend of the incidence of TC

The effect of birth cohort on the risk of TC can be seen in **Figure 1C**. Overall, the younger the birth cohort was, the greater the risk of TC was, and the risk of the cohort in 2005-2014 was almost 2.5 times that of the cohort in 1945~1954. In addition, similar to the period trend, since the birth cohort after 1945~1954, the risk in female has changed from higher to lower than that in male, and the difference in risk ratio has a further widening trend. There was no significant difference in the cohort trend among different SDI countries, but the risk in high SDI countries did not increase with the change of the birth cohort from 1980~1989 to 1995~2004. It is worth mentioning that the birth cohort has the greatest effect on the risk of TC in middle SDI countries, where the relative risk of the 2005~2014 cohort was almost 4 times that of the 1945~1954 cohort, and the relative risk of male was even as high as 8 times.

### The long term trend of age-specific incidence of TC

**Figure 2:**
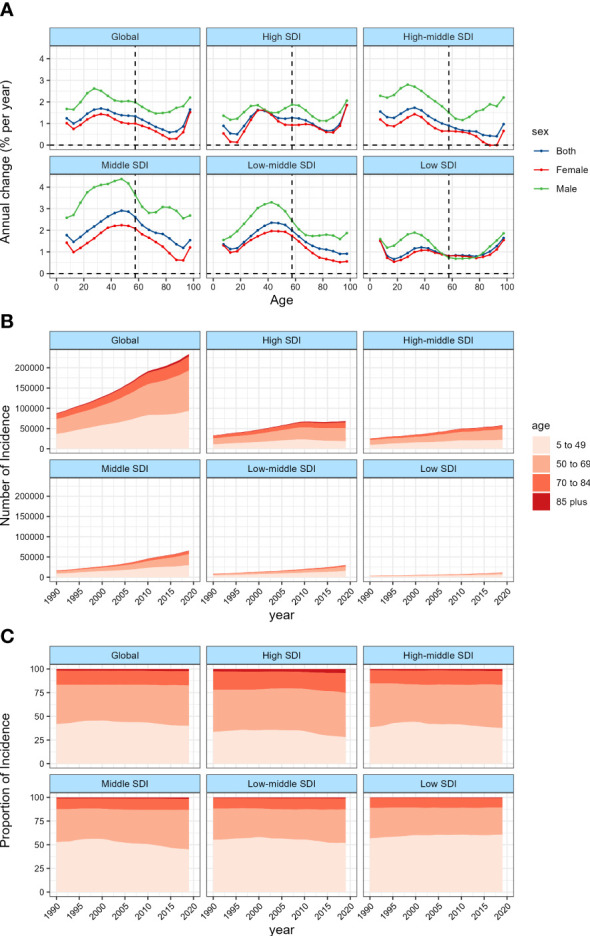
Local drifts of TC incidence and age distribution of TC incidence by SDI quintiles, 1990−2019. **(A)** Local drifts of TC incidence (estimates from APC models) for 19 age groups (5~9 to 95 plus years), 1990~2019. The dots and shaded areas indicate the annual percentage change of incidence (% per year) and the corresponding 95% CIs. **(B)** Temporal change in the absolute cases of TC incidence across age groups, 1990~2019. **(C)** Temporal change in the relative proportion of TC incidence across age groups, 1990~2019.

The annual percentage change of incidence of TC in different age groups was shown in **Figure 2A** and **Supplementary Table 3**. Overall, the incidence was on the rise in all age groups. The people aged 30~34 years old has the largest increase in incidence (1.69% per year, that is, the overall incidence has increased by more than 50% in the past 30 years). The long term trend of age-specific incidence can be roughly divided into 4 stages. In those aged 5~9 years old, the upward trend of TC incidence weakened slightly with the increase of age, and the upward trend increased significantly in those aged 10~34 years old. In those aged 35~84 years old, the upward trend of incidence gradually weakened, and at the age of 85~100 years old, the upward trend increased again. The trend of incidence in different genders was roughly the same, but the incidence growing in male was significantly faster than that in female. The incidence increased the most in middle SDI countries, with an average annual percentage change of 2.91% in people aged 45~49 years old, and 4.38% in male. In contrast, the incidence increased the lowest in high-middle SDI countries among which the increase in the incidence of female aged 85~94 years old was even close to zero.

**Figures 2B, C** and **Supplementary Tables 4**, **5** showed the long term trend of the age distribution of TC. Since 2010, the increasing of TC incidence in all age groups worldwide has slowed down. This change was mainly attributed to the high and high-middle SDI countries, especially in the high SDI countries, the incidence of TC has even begun to decline since 2010, while the incidence in middle to low SDI countries has continued to grow rapidly. Overall, the global age distribution of TC did not change much, the proportion of people over 45 years old increased slightly, while the proportion of people under 45 years old, especially under 20 years old, decreased. However, after stratifying by SDI, we found that this trend was more significant in high and middle SDI countries, while low SDI countries were contrary to the global trend, that is, the new cases transitioned from people over 45 years old to people under 45 years old.

### The trend of TC incidence in various supranational and geographical areas

**Figure 3:**
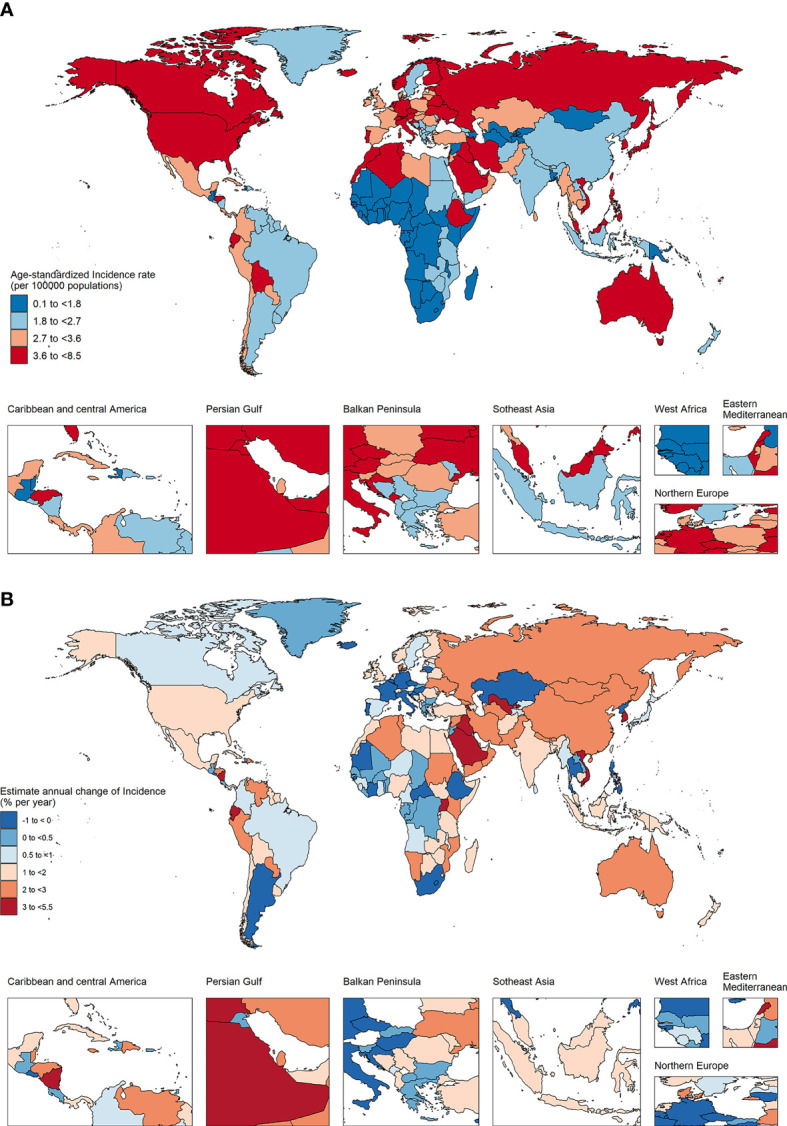
The age-standardized incidence in 2019 **(A)** and net drift of incidence during 1990~2019 **(B)** for TC in 204 countries and territories **(A)** World map of age-standardized incidence for TC in 2019, **(B)** World map of net drifts for TC incidence, ie, estimated annual percentage change of incidence from age-period-cohort model. Net drift captures components of the trends attributable to calendar time and successive birth cohorts.

As shown in **Figure 3** and **Table 1**, the global age-standardized incidence rate (ASIR) of TC in 2019 was 2.83 per 100,000 person-year, which was 40.65% higher than that in 1990. Among the 5 SDI levels, the ASIR in the high SDI countries was the highest, and decreased successively with the decrease of SDI. From 1990 to 2019, the ASIR of TC in the 5 SDI countries all showed a significant upward trend, with the largest upward trend in the middle SDI countries, followed by the middle-low SDI countries.

**Table 1:** Trends in TC incidence across socio-demographic index quintiles, 1990−2019.

Location	Case in 1990	Case in 2019	Case percent change	ASR in 1990	ASR in 2019	ASR percent change	AAPC	Netdrift
Global	87583 (82236, 92717)	233847 (211637, 252807)	167 (143.16, 191.74)	2.01 (1.9, 2.12)	2.83 (2.56, 3.06)	40.65 (28.06, 53.25)	1.11 (0.99, 1.22)	1.24 (1.18, 1.3)
High SDI	32896 (31785, 33893)	68405 (62053, 74858)	107.95 (89.78, 126.98)	3.4 (3.28, 3.5)	4.59 (4.17, 5.03)	35.2 (23, 47.95)	0.95 (0.8, 1.1)	1.15 (1.01, 1.3)
High-middle SDI	25516 (23779, 26871)	57965 (52409, 64001)	126.85 (105.22, 153.44)	2.28 (2.13, 2.4)	3.06 (2.76, 3.38)	34.01 (21.31, 49.77)	0.9 (0.74, 1.06)	1.06 (0.99, 1.14)
Middle SDI	16647 (15141, 18610)	65559 (57599, 73072)	286.41 (228.36, 347.47)	1.31 (1.2, 1.51)	2.49 (2.19, 2.77)	85.95 (55.24, 112.69)	2.2 (2.12, 2.29)	2.22 (2.1, 2.33)
Low-middle SDI	8603 (7223, 10106)	30107 (25984, 33694)	249.25 (186.48, 317.2)	1.13 (0.97, 1.33)	1.91 (1.66, 2.13)	69.02 (39.47, 97)	1.83 (1.71, 1.96)	1.7 (1.57, 1.83)
Low SDI	3872 (2923, 5044)	11680 (9586, 13741)	195.15 (126.75, 296.73)	1.23 (0.98, 1.57)	1.63 (1.34, 1.9)	29.18 (1.83, 64.62)	0.97 (0.86, 1.09)	0.97 (0.74, 1.2)
High-income Asia Pacific	6725 (6336, 7583)	15660 (13134, 18056)	132.84 (79.79, 172.7)	3.32 (3.13, 3.75)	4.98 (4.19, 5.79)	50.02 (13.18, 76.69)	1.21 (0.85, 1.57)	2.04 (1.6, 2.48)
High-income North America	12626 (12140, 13004)	28296 (24461, 32790)	124.11 (93.09, 159.08)	3.96 (3.82, 4.08)	5.4 (4.64, 6.27)	36.27 (17.26, 57.18)	1.09 (0.9, 1.29)	1.04 (0.94, 1.13)
Western Europe	18052 (16979, 18739)	26217 (22591, 30005)	45.23 (26.22, 66.57)	3.63 (3.41, 3.77)	3.93 (3.4, 4.5)	8.27 (-6.42, 25.1)	0.12 (-0.06, 0.3)	0 (-0.1, 0.1)
Australasia	519 (479, 565)	1738 (1359, 2228)	234.82 (152.83, 336.64)	2.31 (2.13, 2.52)	4.44 (3.47, 5.72)	92.28 (44.16, 151.57)	2.36 (2.14, 2.58)	2.46 (2.06, 2.87)
Andean Latin America	426 (368, 517)	2145 (1659, 2688)	403.24 (267.72, 552.55)	1.76 (1.53, 2.12)	3.63 (2.79, 4.54)	105.68 (50.31, 166.39)	2.56 (2.29, 2.83)	2.56 (2.15, 2.98)
Tropical Latin America	1636 (1566, 1730)	4528 (4246, 5027)	176.67 (159.33, 202.5)	1.53 (1.46, 1.61)	1.82 (1.71, 2.02)	19.13 (11.76, 30.13)	0.54 (0.39, 0.69)	0.66 (0.43, 0.9)
Central Latin America	1798 (1698, 1864)	7183 (6143, 8404)	299.6 (241.22, 365.58)	1.8 (1.69, 1.87)	2.9 (2.49, 3.4)	60.94 (37.76, 87.16)	1.71 (1.5, 1.92)	1.5 (1.29, 1.71)
Southern Latin America	959 (882, 1023)	2002 (1561, 2562)	108.73 (61.71, 171.14)	2.04 (1.88, 2.18)	2.58 (2, 3.31)	26.25 (-2.46, 64.55)	0.85 (0.65, 1.04)	0.72 (0.4, 1.04)
Caribbean	486 (448, 521)	1246 (1045, 1482)	156.53 (113.57, 204.19)	1.7 (1.57, 1.82)	2.43 (2.04, 2.89)	42.66 (18.52, 69.41)	1.31 (1.2, 1.43)	1.29 (0.84, 1.74)
Central Europe	4352 (3970, 4532)	5313 (4618, 6120)	22.09 (5.32, 47.88)	3.04 (2.77, 3.16)	3.09 (2.67, 3.57)	1.57 (-12.7, 23.32)	-0.04 (-0.2, 0.12)	-0.19 (-0.4, 0.02)
Eastern Europe	6113 (5750, 6870)	12257 (10669, 14146)	100.51 (71.31, 131.35)	2.28 (2.15, 2.56)	4.25 (3.7, 4.93)	86.48 (59.67, 115.85)	1.96 (1.43, 2.49)	2.38 (2.22, 2.54)
Central Asia	759 (673, 878)	1467 (1306, 1650)	93.25 (66.59, 127.09)	1.44 (1.27, 1.67)	1.69 (1.51, 1.89)	17.74 (1.55, 37.95)	0.58 (0.35, 0.81)	0.56 (0.1, 1.03)
North Africa and Middle East	3882 (3233, 4451)	19253 (15675, 22281)	396.01 (315.11, 526.13)	1.7 (1.44, 2)	3.46 (2.89, 3.96)	103.12 (67.32, 155.48)	2.45 (2.39, 2.52)	2.44 (2.26, 2.63)
South Asia	7930 (6672, 9896)	31534 (26591, 36439)	297.65 (196.33, 388.6)	1.05 (0.9, 1.32)	1.9 (1.61, 2.19)	81.02 (35.31, 119.85)	2.13 (1.93, 2.33)	1.96 (1.77, 2.14)
Southeast Asia	7200 (5805, 8275)	25581 (20569, 29886)	255.3 (194.76, 330.81)	2.25 (1.86, 2.55)	3.72 (3.01, 4.32)	65.42 (38.6, 96.57)	1.71 (1.62, 1.8)	1.64 (1.51, 1.77)
East Asia	11092 (9401, 13053)	41580 (34751, 50204)	274.86 (193.51, 389.42)	1.07 (0.92, 1.27)	2.11 (1.77, 2.54)	96.16 (53.6, 152.37)	2.34 (2.14, 2.55)	2.41 (2.14, 2.68)
Oceania	54 (42, 70)	162 (114, 220)	199.47 (132.19, 291.07)	1.45 (1.15, 1.86)	1.8 (1.3, 2.4)	24.26 (-3.19, 59.22)	0.79 (0.65, 0.93)	0.68 (-0.95, 2.34)
Western Sub-Saharan Africa	375 (298, 437)	1083 (857, 1325)	188.99 (134.64, 255.34)	0.36 (0.28, 0.42)	0.43 (0.34, 0.52)	20.5 (0.07, 45.72)	0.59 (0.48, 0.7)	0.77 (0.26, 1.29)
Eastern Sub-Saharan Africa	2063 (1400, 2886)	5343 (4114, 6792)	159.02 (77.17, 306.9)	1.94 (1.41, 2.65)	2.19 (1.72, 2.72)	12.5 (-19.22, 60.76)	0.39 (0.28, 0.51)	0.43 (0.09, 0.78)
Central Sub-Saharan Africa	192 (138, 258)	520 (363, 729)	170.83 (96.9, 263.37)	0.72 (0.51, 0.98)	0.8 (0.54, 1.14)	11.19 (-17.7, 45.3)	0.35 (0.21, 0.5)	0.36 (-0.51, 1.23)
Southern Sub-Saharan Africa	345 (296, 380)	739 (633, 859)	114.4 (83.42, 154.82)	1 (0.85, 1.1)	1.13 (0.97, 1.3)	12.83 (-1.85, 30.88)	0.43 (0.34, 0.52)	0.28 (-0.23, 0.79)

Among the 21 geographical areas, the ASIR of High-income North America was the highest, while the ASIR of Western Sub-Saharan Africa was the lowest. The ASIR of TC in all geographical areas except Western Europe showed an upward trend, of which Andean Latin America was the highest, followed by North Australasia.

### The trend of TC incidence in various countries and regions

In 2019, 38 countries and regions had more than 1000 new cases, of which China, United States of America, India and Japan were the four countries with the largest number of new cases, accounting for 42.5% of the global new cases (**Supplementary Table 6**). Among these 38 countries, the incidence showed a downward trend in 8 countries and an upward trend in the rest. The ASIR of Viet Nam was the highest, while the ASIR of Bangladesh was the lowest, and the net drift of Saudi Arabia was the highest, while the net drift of Poland was the lowest. In 2019, the ASIR of 95 countries was higher than the global level, and the ASIR of 10 countries (Saudi Arabia, Palau, Italy, San Marino, Monaco, Republic of Korea, Viet Nam, Honduras, Lebanon, and Iceland) was more than twice that of the global level.

### APC effect in exemplary countries

**Figure 4:**
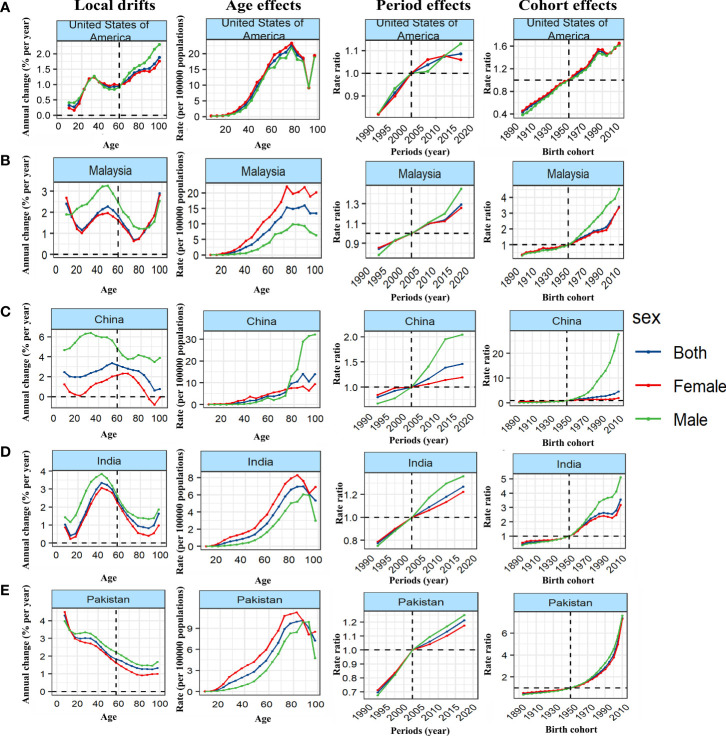
Unfavourable APC effects on exemplar countries across **(A)** high-, **(B)** high-middle, **(C)** middle, **(D)** middle-low and **(E)** low-SDI countries. Local drifts indicate the annual percentage change of incidence (% per year) across five-year age groups (from 5 to 9 to 95 plus years). Age effects are represented by the fitted longitudinal age curves of incidence (per 100,000 person-years) adjusted for period deviations. Period effects are represented by the relative risk of incidence (incidence rate ratio) and computed as the ratio of age-specific rates in each period compared to the referent period. Cohort effects are represented by the relative risk of incidence (incidence rate ratio) and computed as the ratio of age-specific rates in each cohort compared to referent 1950 cohort.

**Figure 5:**
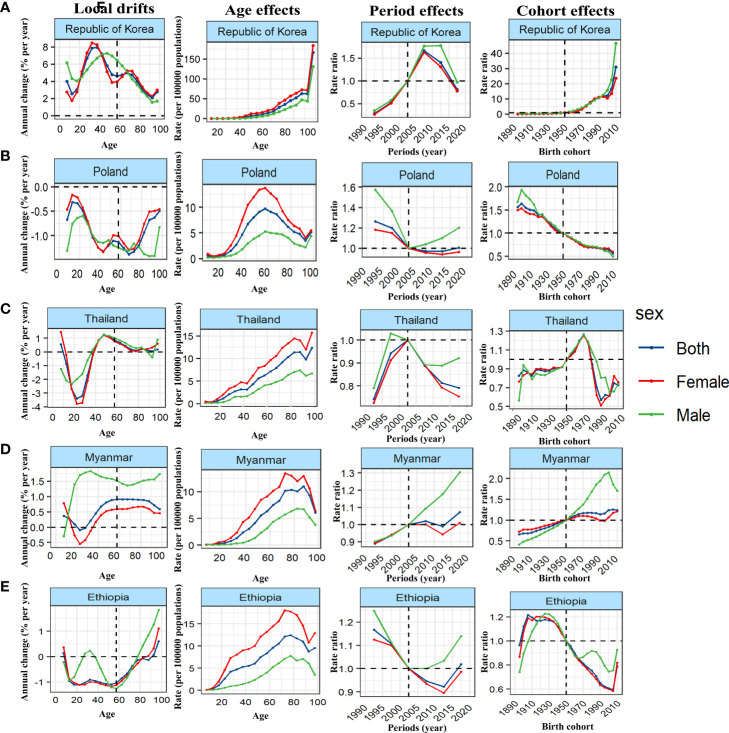
Favourable APC effects on exemplar countries across **(A)** high-, **(B)** high-middle, **(C)** middle, **(D)** middle-low and **(E)** low-SDI countries. Local drifts indicate the annual percentage change of incidence (% per year) across five-year age groups (from 5 to 9 to 95 plus years). Age effects are represented by the fitted longitudinal age curves of incidence (per 100,000 person-years) adjusted for period deviations. Period effects are represented by the relative risk of incidence (incidence rate ratio) and computed as the ratio of age-specific rates in each period compared to the referent period. Cohort effects are represented by the relative risk of incidence (incidence rate ratio) and computed as the ratio of age-specific rates in each cohort compared to referent 1950 cohort.

We further stratified the 38 countries with the highest number of cases by SDI, and took typical countries as an example to describe the APC effect of different countries. United States of America showed a typical age effect of TC incidence in high SDI countries, and showed the unfavorable period and cohort effects (**Figure 4A**; **Supplementary Figures 1**–**4**). In contrast, the incidence of TC in Republic of Korea showed a steady upward trend at all ages and birth cohort, and a relatively favorable downward period effect, that is, the rising risk has been reversed and decreased rapidly since 2007 so that the risk of TC has fallen below the reference time since 2015 (**Figure 5A**). The age and period effect in Japan was highly similar to that in Korea, but its cohort effect was relatively moderate, and the risk of TC did not increase significantly with the birth cohort (**Supplementary Figures 2**–**4**). It is noteworthy that the cohort effect in Japan showed obvious gender disparities that male showed an unfavorable upward cohort effect far exceeding female, while Canada showed a period effect similar to Korea and a cohort effect similar to Japan (**Supplementary Figures 3**, **4**). The age effect of Germany and France was similar to that of other high SDI countries, but the age of highest risk in Germany was significantly smaller than that in most high SDI countries, and France did not show significant gender disparities (**Supplementary Figure 2**). What is more interesting is the period effect of the two countries. France showed a period effect in which the risk increased first and then decreased, so that the risk reached its peak in 2003, and before that, there was a significant gender disparity, while in Germany, it was completely opposite, that is, the period effect of risk decreased first and then increased, and the gender disparity did not appear until after 2003 (**Supplementary Figure 3**). In terms of cohort effect, the two countries were quite similar, that is, there was a diametrically opposite trend of cohort effect between the genders (**Supplementary Figure 4**). In addition, although the age effect of a province of China—Taiwan was similar to most high SDI countries, its period effect showed obvious gender disparity since 2003 and the disparity was further expanding. This gender disparity can also be seen in the cohort effect which exhibited an inverted U-shaped curve that increased first and then decreased from 1950 to 1990 (**Supplementary Figures 2**–**4**).

Malaysia showed the typical unfavorable APC effect of high-middle SDI countries, that is, the incidence of TC increased with the changes of age, period and birth cohort, and the gender disparity increased gradually, which was similar to Saudi Arabia, Turkey and Ukraine (**Figure 4B**; **Supplementary Figures 5**–**8**). It should be pointed out that although the general trend of Russian Federation was similar to that of the above countries, it showed a completely different gender disparity, that is, in the cohort and period effects, the risk of TC in female has changed from lower to higher than that in male (**Supplementary Figures 2**–**4**). On the contrary, Poland showed a favorable period-cohort effect, that is, with the change of period and birth cohort, the risk of TC decreased (**Figure 5B**). Italy showed a special age-cohort effect that male and female showed opposite cohort effect, and female showed a much more unfavorable period effect than male (**Supplementary Figures 6**–**8**). Among the 13 middle SDI countries with high incidence, eleven countries showed an unfavorable APC effect similar to China, and China also showed a more significant gender disparity that male suffered a more unfavorable age-period effect than female (**Figure 4C**; **Supplementary Figures 9**–**12**). Thailand and Philippines were different from these countries which exhibited a favorable APC effect, that is, with the change of birth cohort, the TC risk decreased in a fluctuating manner (**Figure 5C**). The middle-low SDI countries were represented by India, which showed an unfavorable APC effect, and the same effect can also be seen in Bangladesh and Morocco (**Figure 4D**; **Supplementary Figures 13**–**16**), while Myanmar showed a relatively favorable APC effect, mainly due to the decrease in TC risk from 2007 to 2012 and its cohort in 1990 (**Figure 5D**). There were only two low SDI countries with more than 1000 cases in 2019, but they also showed different APC effects. Both Ethiopia and Pakistan showed that the incidence rate of people under 78 years old increased with age, while that of people over 78 years old decreased with age. In the cohort and period effect, Pakistan showed an obvious unfavorable upward trend of risk (**Figure 4E**; **Supplementary Figures 17**–**20**), while Ethiopia showed the opposite trend (**Figure 5E**).

## Discussion

### The age effect

With the increase of age, the accumulation of risk factors and the increase of environmental exposure, it is an indisputable fact that people are more likely to suffer from cancers, and the accelerated transformation of global aging may aggravate this trend (14). APC analysis of the incidence of TC between China and the United States reported that the incidence peaked at 55~59 years old (15), while another APC study aimed at Chinese showed that young people aged 10~24 years would be the main population for the increase of TC incidence in the next 20 years (16). From the perspective of age effect, basically, the incidence of TC all over the world is unfavorably increasing with age, especially for the elderly over 90 years old. Given that such people account for a relatively small proportion and receive additional attention from medical care, it is necessary to further confirm the real reason for its rising incidence of TC.

Interestingly, we noticed that among these exemplary countries, the peak of age effect in Poland, Italy, Germany, France and Ukraine occurred around 60 years old, significantly earlier than in other countries, suggesting that they have a unique genetic predisposition. Since these five countries are all European countries, genotypes should be considered first. Previous studies have reported that there is a gene mutation from a common ancestor such as BRCA1mutation c.5266dupC, and BRCA1 is associated with TC incidence (17, 18). However, gene mutations do not perfectly explain the age effect. United Kingdom, also a typical European country, does not fit the above hypothesis. The possible explanation is epigenetic alterations caused by differences in iodine intake, which has been proposed as an epigenetic regulator for TC (19), and United Kingdom is one of the few non-iodine deficient countries in Europe (20). These hypotheses need to be confirmed in further prospective studies.

### The period effect

The true reason for the rapid increase in the incidence of TC has long been controversial by researchers and some believe that overdiagnosis should be the cause (21, 22). Overdiagnosis refers to early cancer patients detected by screening, some of which may never develop to the advanced stage with clinical symptoms (23). These patients are overdiagnosed, which caused 90% of new cases in South Korea, 70% in the United States, Italy, France and Australia, and about 50% in Japan and England (24). The evidence supporting the overdiagnosis hypothesis includes: (1) The increase in the incidence of TC is accompanied by a stable or decreasing death rate (16, 25, 26); (2) The detection of small-sized tumors increased (27, 28); (3) The number of new cases of TC is closely related to the number of examinations by ultrasound, CT and other diagnostic techniques (29, 30). However, there are also evidences that challenge the view of overdiagnosis: (1) The detection of large tumors is also increasing, not only small tumors (31, 32); (2) Although the overall death rate remained stable, the mortality of advanced tumors really increased (33, 34); (3) Molecular genetic characteristics of TC such as BRAF and RAS mutations are changing with time (35).

The period effect in our APC model provides a new perspective for this controversy. The increase in the risk of TC has slowed significantly since 2007 and this is mainly attributed to high SDI and high-middle SDI countries, which is basically consistent with the timing of changes of guidelines in South Korea and the United States (36, 37). From the analysis of period effect in Korea, the risk of TC has dropped sharply since 2007, which provides strong support for the overdiagnosis hypothesis. However, the risk of TC in the United States has not decreased since the revision of the guidelines in 2009. The distinct effects of the guideline revisions between South Korea and the United States deserve policy makers and clinical practitioners to carefully examine the underlying causes and take appropriate measures. In addition, we can also see that the risk of TC in middle and lower SDI countries is still rising rapidly, indicating that their understanding of overdiagnosis is insufficient and need to further promote the reform the guidelines of diagnosis and treatment for TC (38).

### The cohort effect

Ionizing radiation is one of the definite risk factors for TC, including radiation pollution caused by nuclear facilities and radiation produced by medical means, in which iodine radiation pollution has a dose-effect relationship with the risk of TC (39–41). Whether it is nuclear contaminated or iatrogenic radiation, children and adolescents seem to be susceptible to this risk factor. Previous studies have reported a rapid increase in the incidence of TC in children and adolescents (42, 43). However, in view of the same rising incidence in adults, we believe that the proportion of TC in children and adolescents in the total population may be more reasonable for exploring the influence of its specific risk factors, so we investigated the countries—Ukraine and Japan, where the famous Chernobyl and Fukushima accidents are located in, and speculated the effect of ionizing radiation on the risk of TC in children and adolescents through the cohort effect.

From the results, the birth cohort in Ukraine from 1965 to 1980 and the birth cohort in Japan from 1990 to 2000 does not show a significant increase in risk, and The proportion of TC in children and adolescents in the total population in these two countries has also been steadily declining. The negative results can be attributed to three reasons: (1) The incubation period of ionizing radiation exposure to TC is 5~10 years (44), and the time after the Fukushima accident may not be enough to induce a large number of TC; (2) Due to the lack of state and provincial data, we can only get data on the incidence of Japan and Ukraine rather than those of Fukushima and Pripyat; (3) The periods of ionizing radiation exposure overlapped with the spread of overdiagnosis so it is difficult to separate the effects of these two factors. In summary, the impact of nuclear contamination accidents on the incidence of TC at the national level may be very limited, but we still need to pay attention to the impact of iatrogenic factors such as dental X-ray or CT (45).

### Sex and social demographic status

The traditional view is that TC is more common in women, and this gender disparity may be related to the factors of obstetrics and gynecology, the high availability medical care and the level of sex hormones (46–49). Our study found that although the incidence of TC in female is still much higher than that of male, male show a worrying pattern of incidence growth. Since 2002, the risk of TC in male has exceeded that of female, and this gap has further increased in middle and higher SDI countries, which has been confirmed in previous studies (50). What is more vigilant is that many studies have pointed out that the prognosis of male is worse than that of female (51, 52), which suggests that there may be biological factors other than overdiagnosis in the increased incidence of TC in male. So it is necessary to pay close attention to the incidence and prognosis trend of TC in male and formulate corresponding strategies.

Previous studies have reported that the incidence of TC was positively correlated with SDI (1). It is generally believed that with the development of economy, the improvement of living standards and the enhancement of health care awareness, physical examination will become an urgent health need for people, thus accelerating the discovery of TC. However, we can find from our results that although high SDI countries do have the highest ASR, there is the greatest upward trend in middle and middle-low SDI countries, which confirms the previous conclusion that overdiagnosis is also noteworthy in middle- and low-income countries (53). It is suggested that when developed countries are gradually aware of the adverse effects of overdiagnosis, this concept should be transferred to other countries to reduce their burden of TC. When making such comparisons, we must note that the data quality varies from country to country, and previous studies have used rating systems to assess the quality of data available in each country for our reference (54).

## Limitations

The limitations of this study include: (1) As a population-based analysis, we need to be very vigilant against ecological fallacies, and when explaining the results, we can only put forward the scientific hypothesis of the causality of the epidemic trend based on the existing data and previous literature rather than subjectively infer it; (2) There may be great discrepancies in the incidence among different pathological types of TC, which is not reflected in the GBD database; (3) There may be significant differences in incidence rate among different regions of the same country, especially in countries with a large population and a vast territory. GBD does not record in detail the uneven development within the country. (4) As a study based on APC model, we provided clues to the long-term trend and some causes of TC from a dynamic point of view, but the effects of many suspicious risk factors such as iodine intake and endocrine chemical disruptors have not been fully investigated.

## Conclusion

The trend of rapid increase in the incidence of TC has begun to slow in recent years, which mainly due to the awareness of the impact of overdiagnosis in developed countries. However, the global incidence of TC has obvious gender and regional/national heterogeneity: Male show a more worrying pattern of incidence growth than female; The incidence of TC is still rising sharply in middle and lower SDI countries; After the revision of the guidelines, The risk of TC in South Korea has been suppressed, while the United States has had little effect. Policy makers should tailor specific local strategies to the risk factors of each country to further reduce the burden of TC.

## Data availability statement

Publicly available datasets were analyzed in this study. This data can be found here: http://ghdx.healthdata.org/gbd-results-tool.

## Author contributions

LX and ZXC conceived and designed the study. CFW supervised the study. LX and CZX performed the statistical analysis. XW offered methodological guidance and data visualization. All authors contributed to the article and approved the submitted version. ZXC drafted the manuscript. All authors revised the report.
