# Supplementary material for: Global thyroid cancer incidence trend and age-period-cohort model analysis based on Global Burden of Disease Study from 1990 to 2019
Source: Front Endocrinol (Lausanne). 2023 Apr 12;14:1133098. doi: 10.3389/fendo.2023.1133098 (PMC10130642; doi:10.3389/fendo.2023.1133098)
Supplement: Supplementary file 7 [file DataSheet_7.docx]

Supplementary Figures


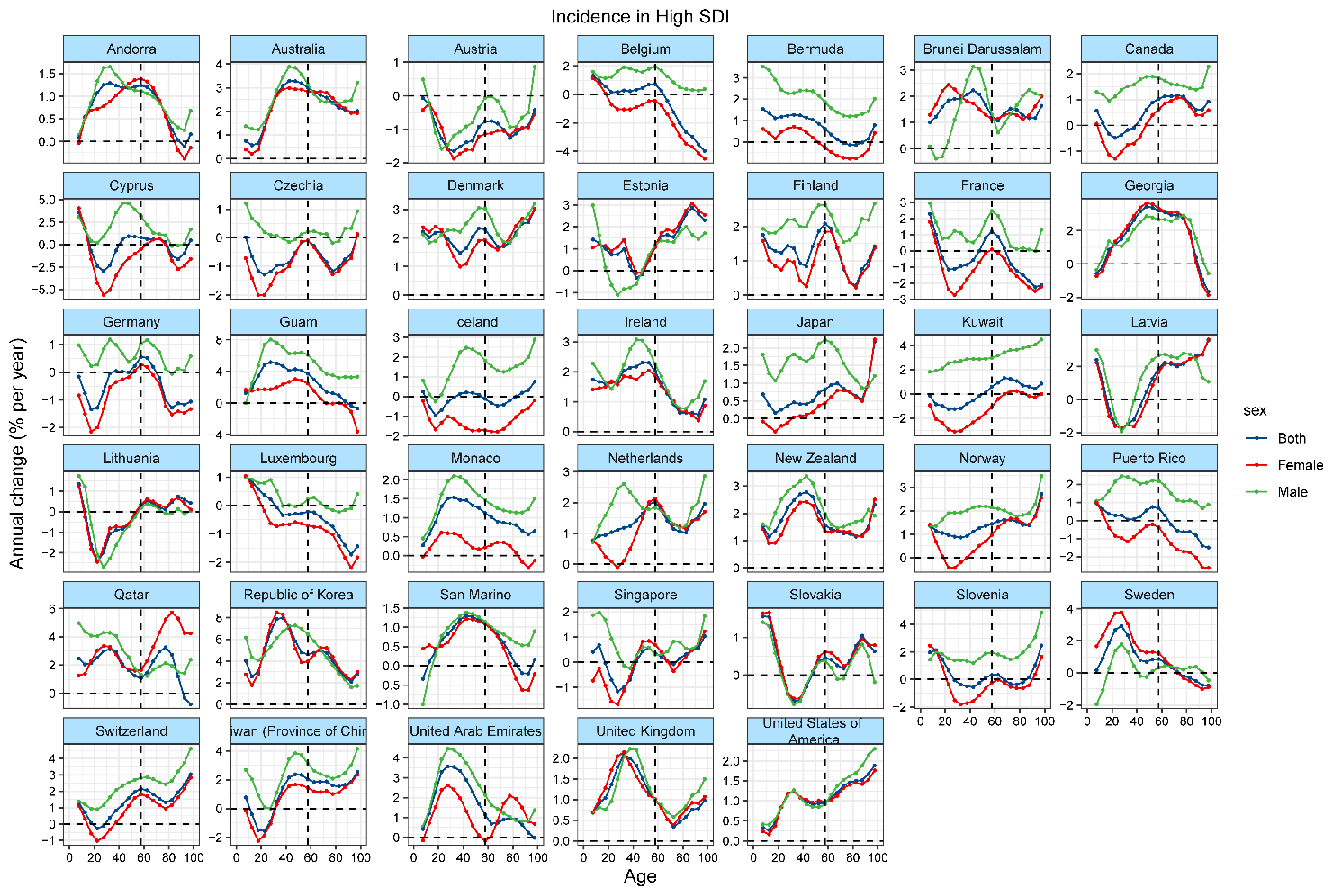


**Supplementary Figure 1. Incidence local drift in high SDI countries.** The local drifts of thyroid cancer incidence in high-SDI countries, 1990-2019. Local drifts of thyroid cancer incidence (estimates from age-period-cohort models) for 19 age groups (5−9 to 95 plus years), 1990−2019.


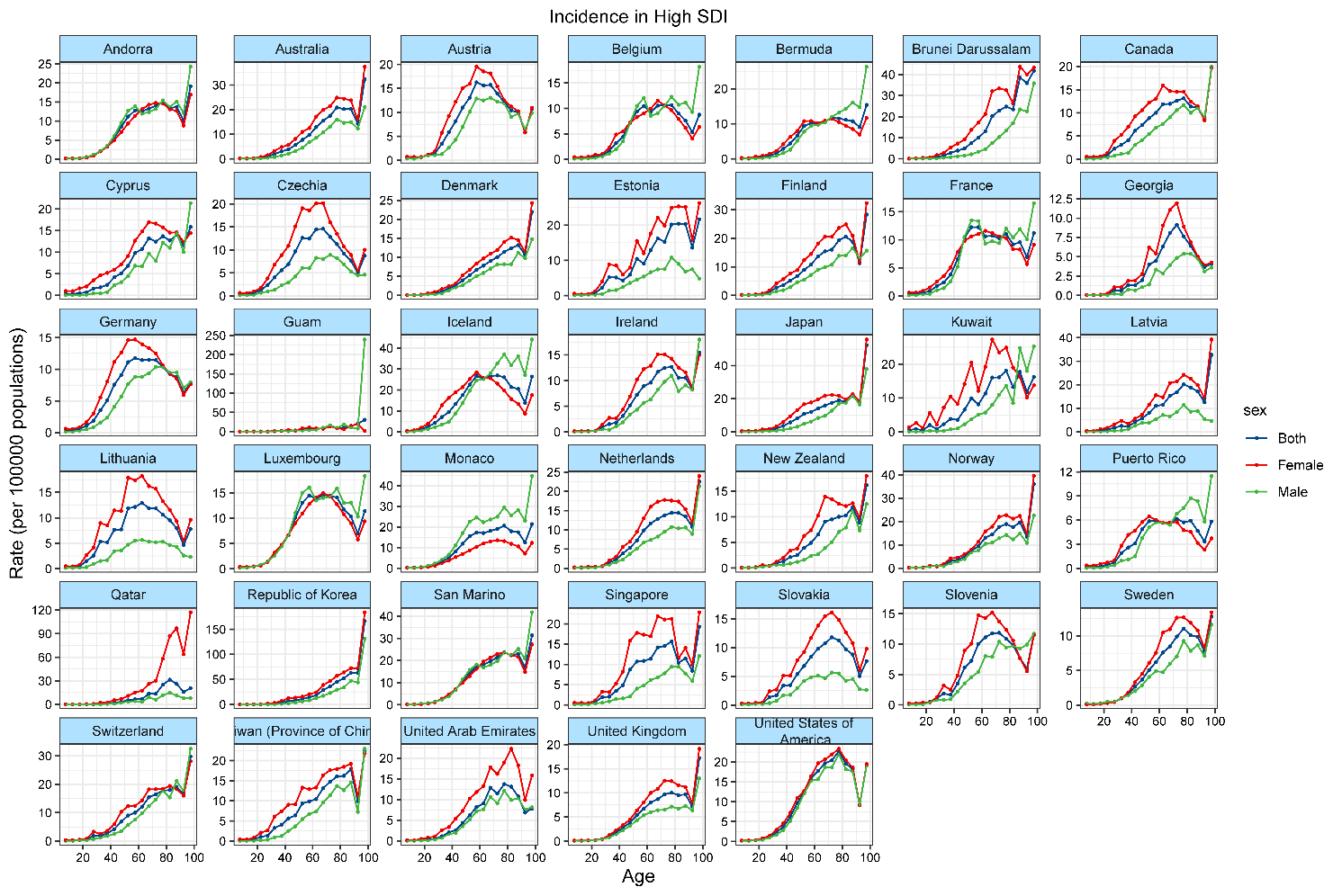


**Supplementary Figure 2. Incidence age effect in high SDI countries.** Age effects on thyroid cancer incidence in high-SDI countries. Age effects are shown by the fitted longitudinal age curves of incidence (per 100,000 person-years) adjusted for period deviations. (B) Period effects are shown by the relative risk of incidence (incidence rate ratio) and computed as the ratio of age-specific rates from 1990−1994 to 2015−2019 (2000−2005 as the referent period).


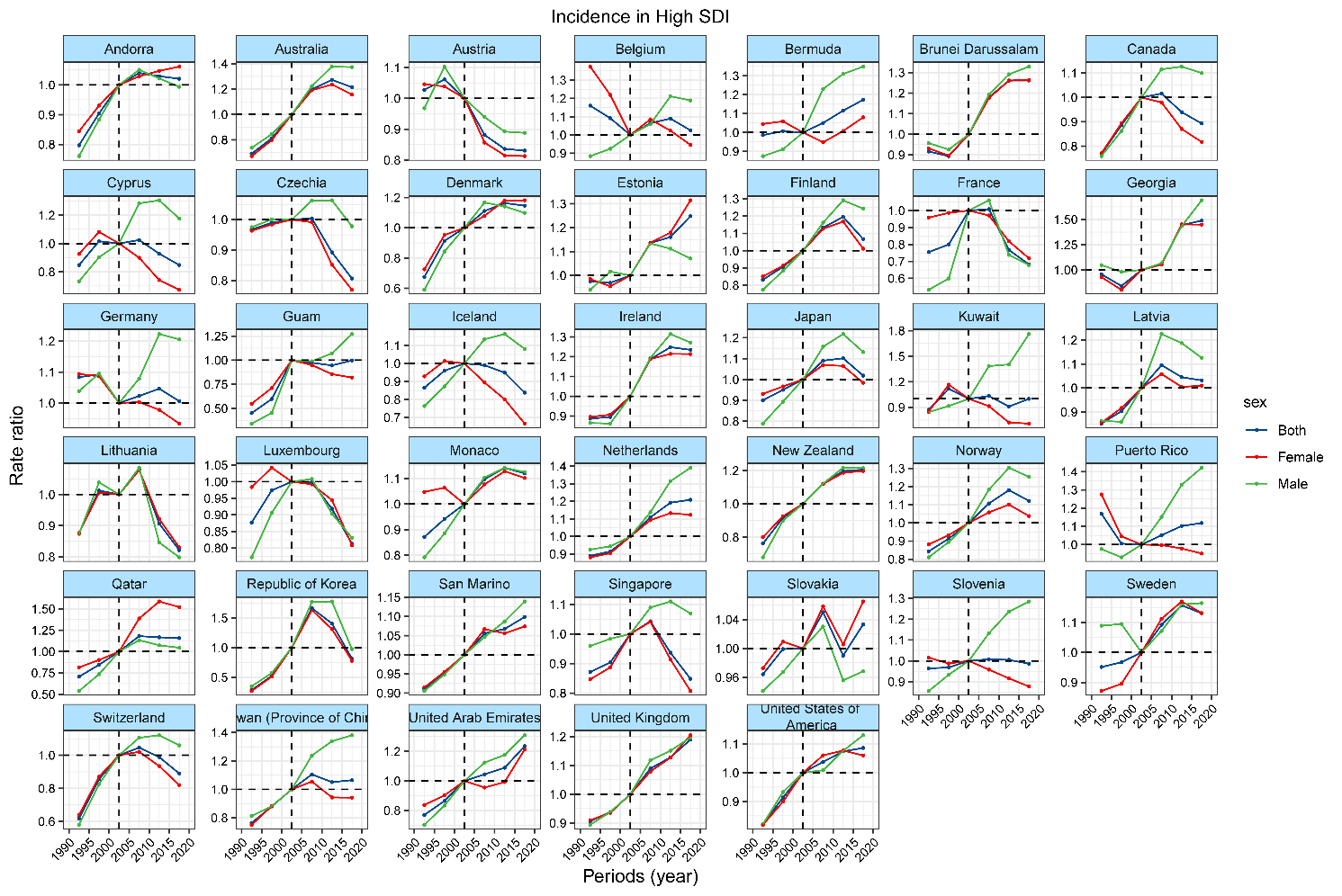


**Supplementary Figure 3. Incidence period effect in high SDI countries.** Period effects on thyroid cancer incidence in high-SDI countries. Period effects are shown by the relative risk of incidence (incidence rate ratio) and computed as the ratio of age-specific rates from 1990−1994 to 2015−2019 (2000−2005 as the referent period).


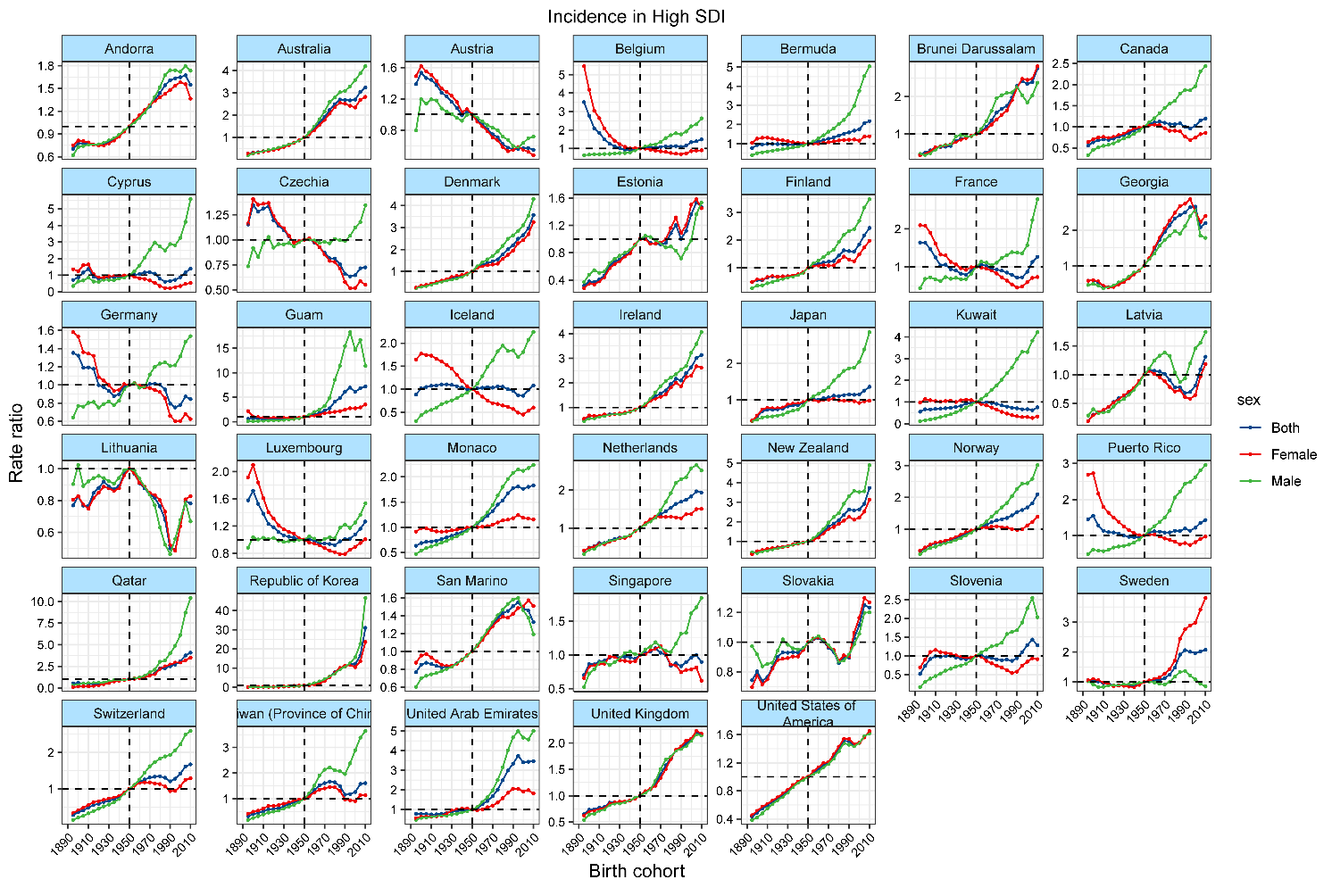


**Supplementary Figure 4. Incidence cohort effect in high SDI countries.** Cohort effects on thyroid cancer incidence in high-SDI countries. Cohort effects are shown by the relative risk of incidence and computed as the ratio of age-specific rates from the 1895 cohort to the 2010 cohort, with the referent cohort set at 1950.


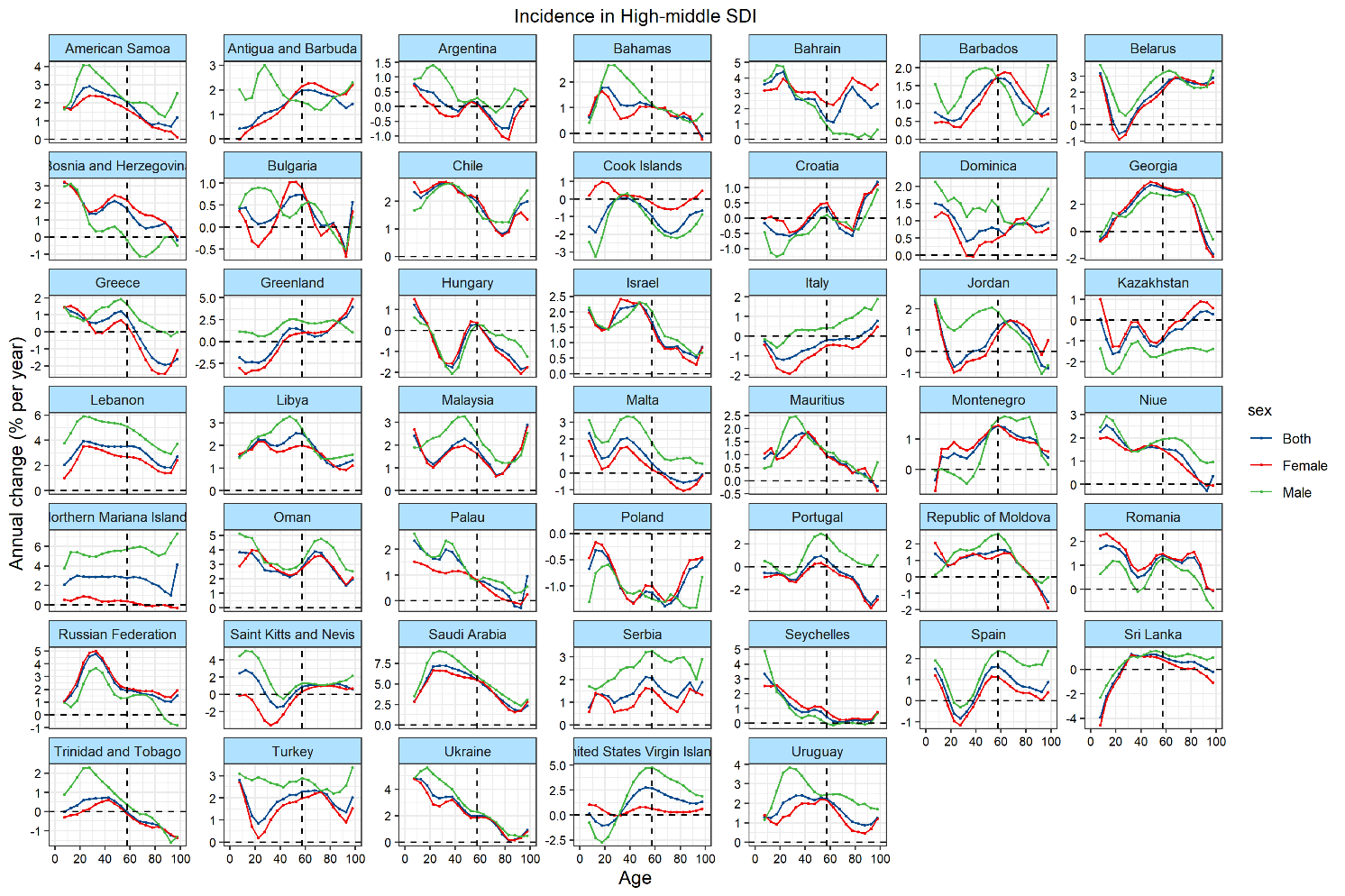


**Supplementary Figure 5. Incidence local drift in high-middle SDI countries.** The local drifts of thyroid cancer incidence in high-middle-SDI countries, 1990-2019. Local drifts of thyroid cancer incidence (estimates from age-period-cohort models) for 19 age groups (5−9 to 95 plus years), 1990−2019.


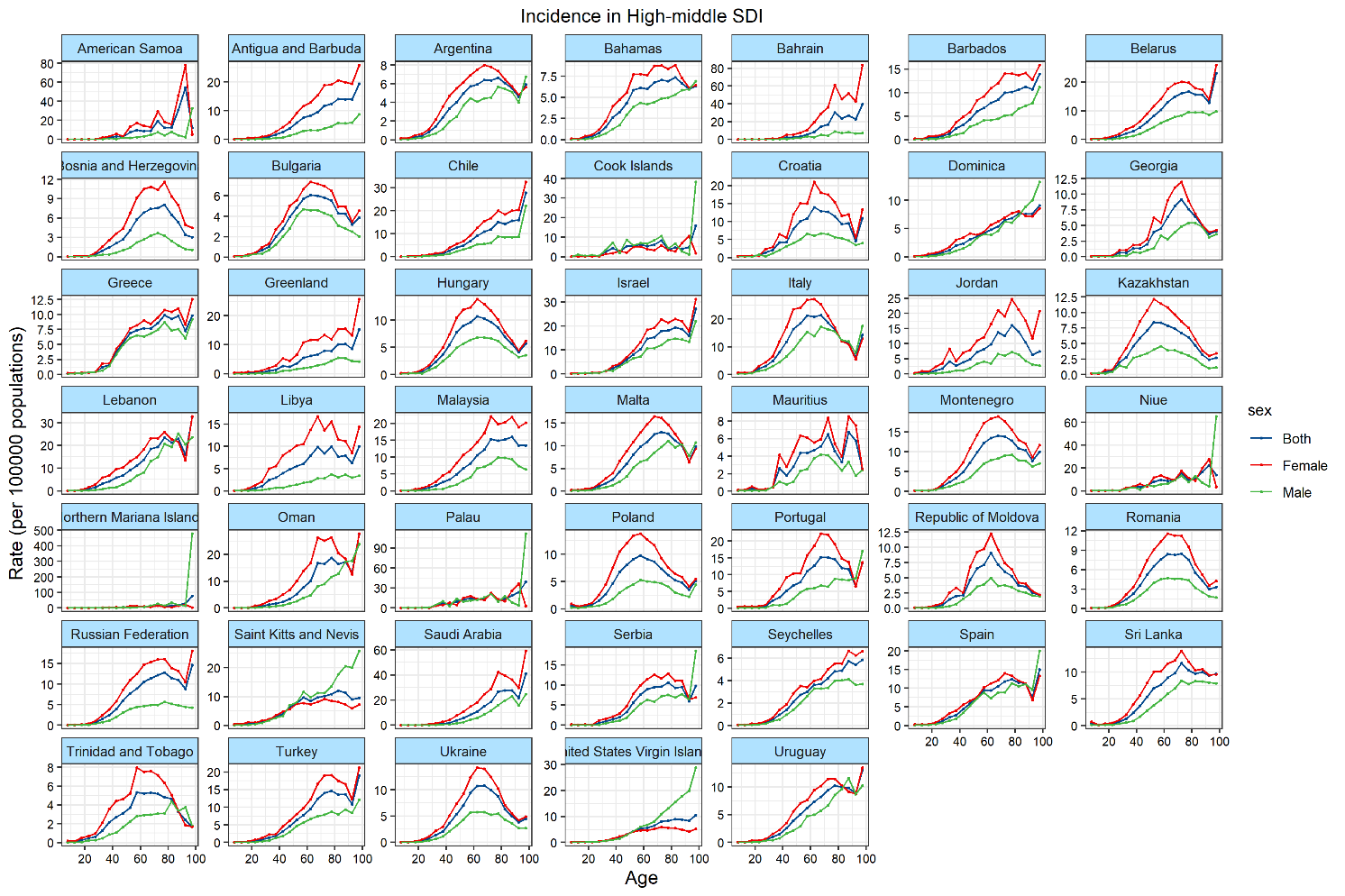


**Supplementary Figure 6. Incidence age effect in high-middle SDI countries.** Age effects on thyroid cancer incidence in high-middle-SDI countries. Age effects are shown by the fitted longitudinal age curves of incidence (per 100,000 person-years) adjusted for period deviations. (B) Period effects are shown by the relative risk of incidence (incidence rate ratio) and computed as the ratio of age-specific rates from 1990−1994 to 2015−2019 (2000−2005 as the referent period).


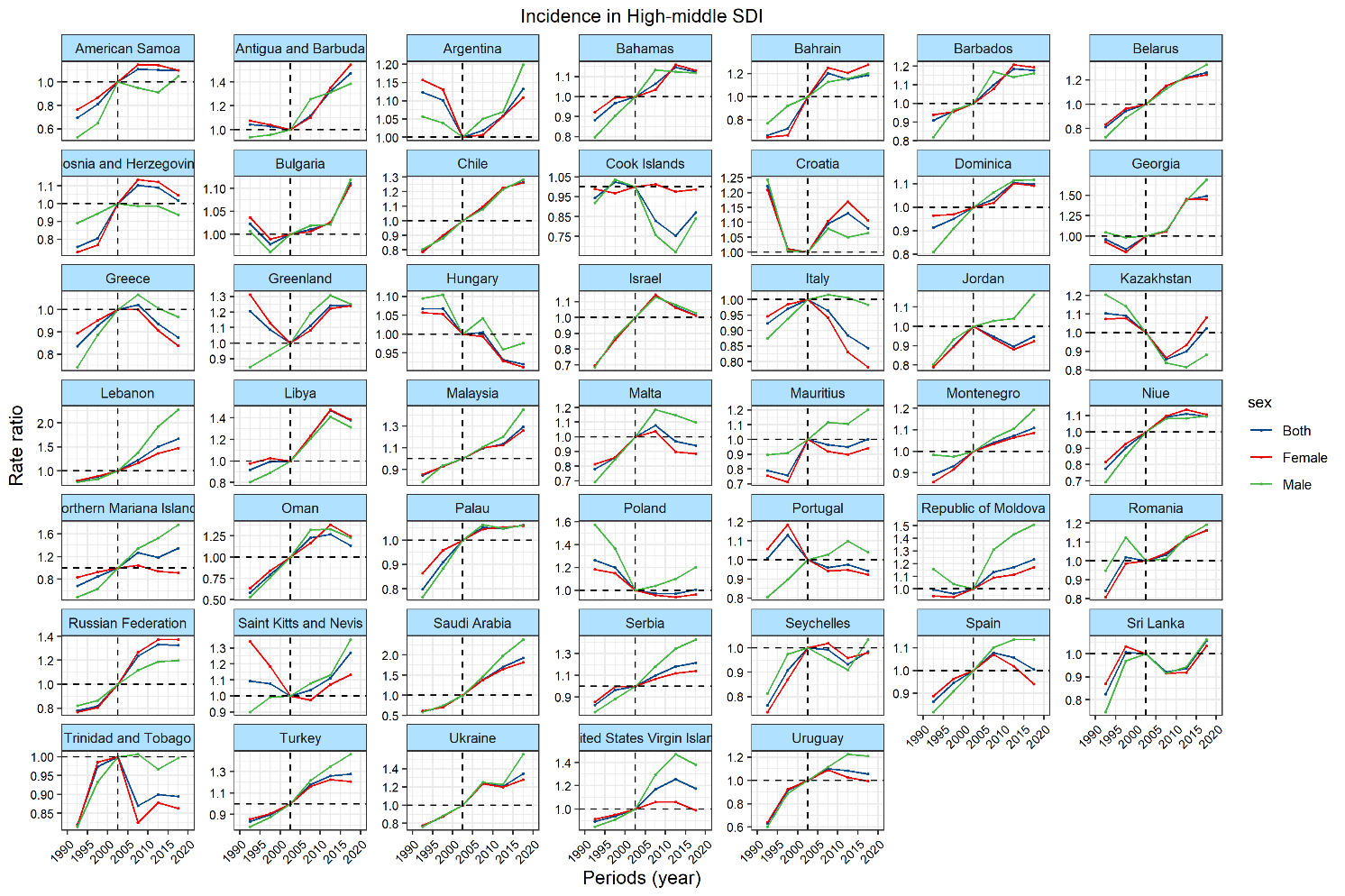


**Supplementary Figure 7. Incidence period effect in high-middle SDI countries.** Period effects on thyroid cancer incidence in high-middle-SDI countries. Period effects are shown by the relative risk of incidence (incidence rate ratio) and computed as the ratio of age-specific rates from 1990−1994 to 2015−2019 (2000−2005 as the referent period).


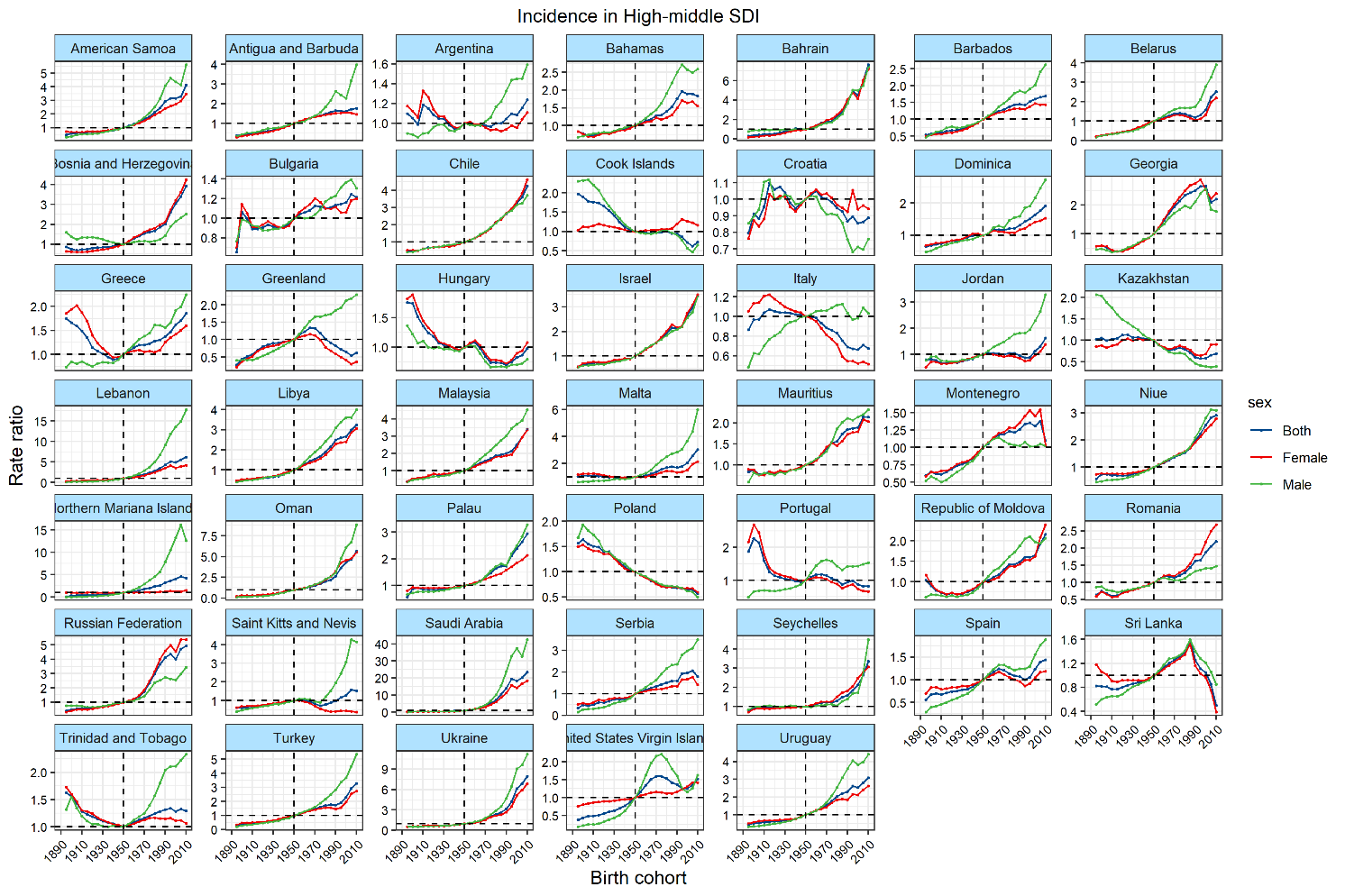


**Supplementary Figure 8. Incidence cohort effect in high-middle SDI countries.** Cohort effects on thyroid cancer incidence in high-middle-SDI countries. Cohort effects are shown by the relative risk of incidence and computed as the ratio of age-specific rates from the 1895 cohort to the 2010 cohort, with the referent cohort set at 1950.


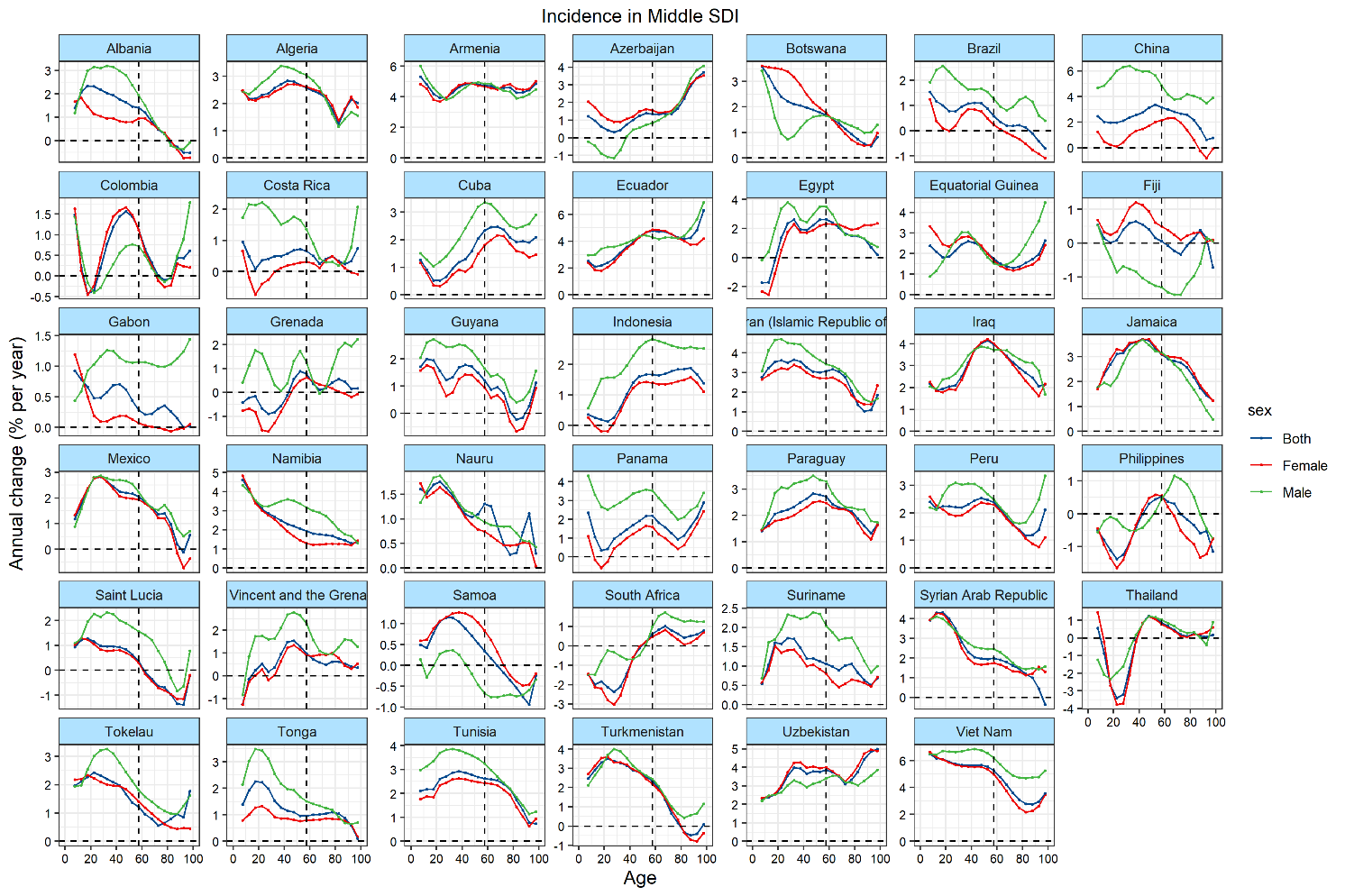


**Supplementary Figure 9. Incidence local drift in middle SDI countries.** The local drifts of thyroid cancer incidence in middle-SDI countries, 1990-2019. Local drifts of thyroid cancer incidence (estimates from age-period-cohort models) for 19 age groups (5−9 to 95 plus years), 1990−2019.


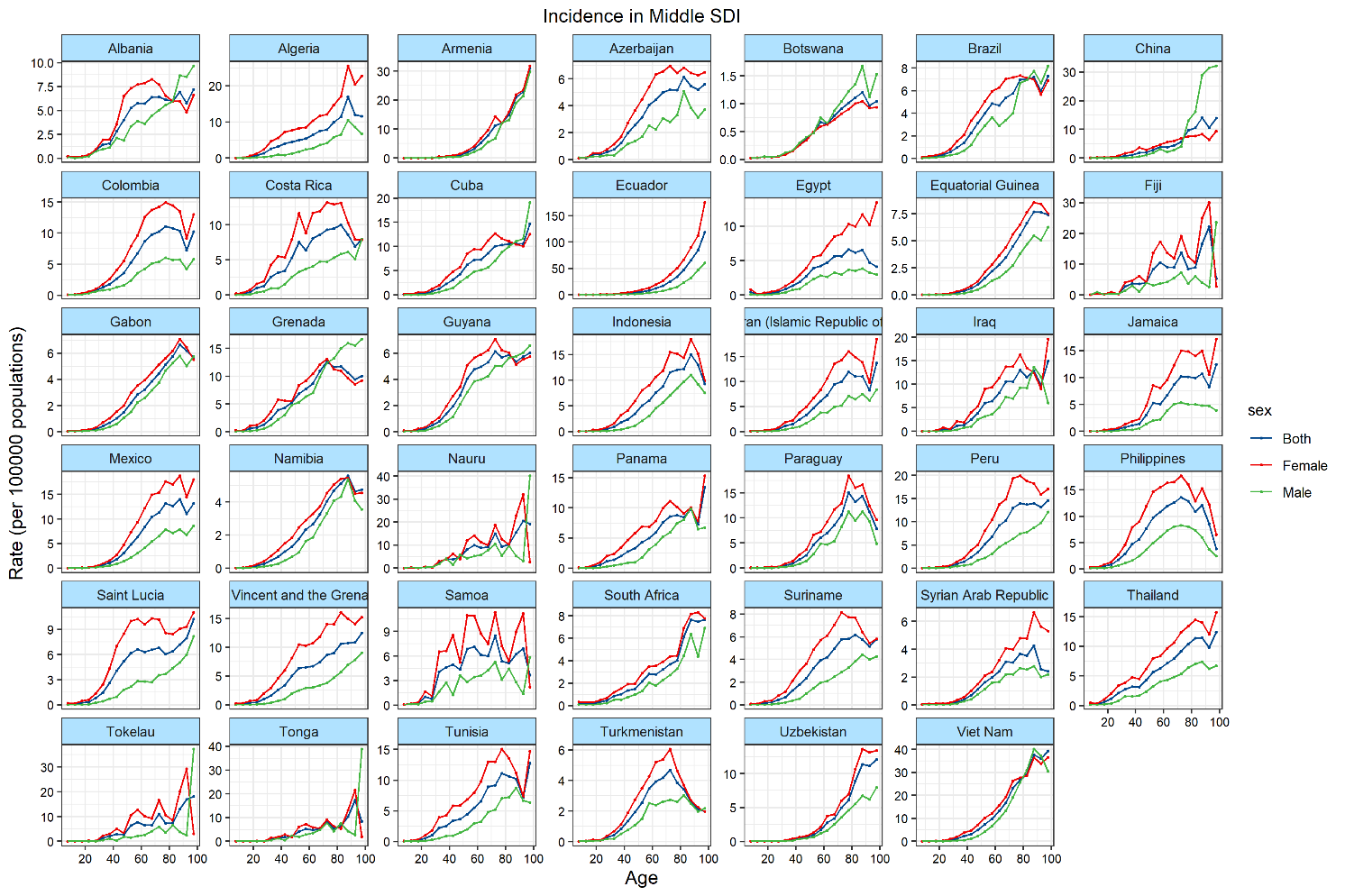


**Supplementary Figure 10. Incidence age effect in middle SDI countries.** Age effects on thyroid cancer incidence in middle-SDI countries. Age effects are shown by the fitted longitudinal age curves of incidence (per 100,000 person-years) adjusted for period deviations. (B) Period effects are shown by the relative risk of incidence (incidence rate ratio) and computed as the ratio of age-specific rates from 1990−1994 to 2015−2019 (2000−2005 as the referent period).


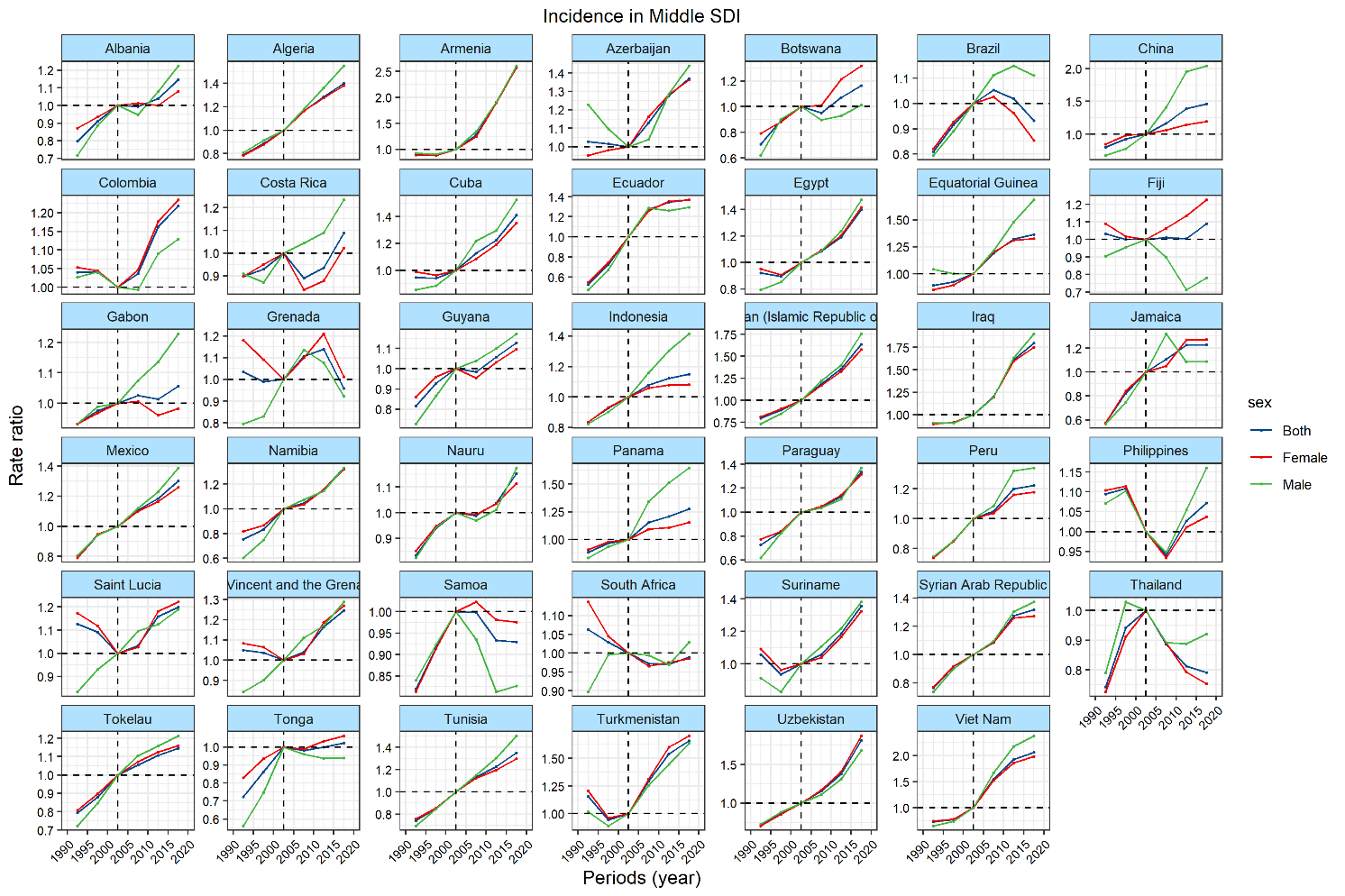


**Supplementary Figure 11. Incidence period effect in middle SDI countries.** Period effects on thyroid cancer incidence in middle-SDI countries. Period effects are shown by the relative risk of incidence (incidence rate ratio) and computed as the ratio of age-specific rates from 1990−1994 to 2015−2019 (2000−2005 as the referent period).


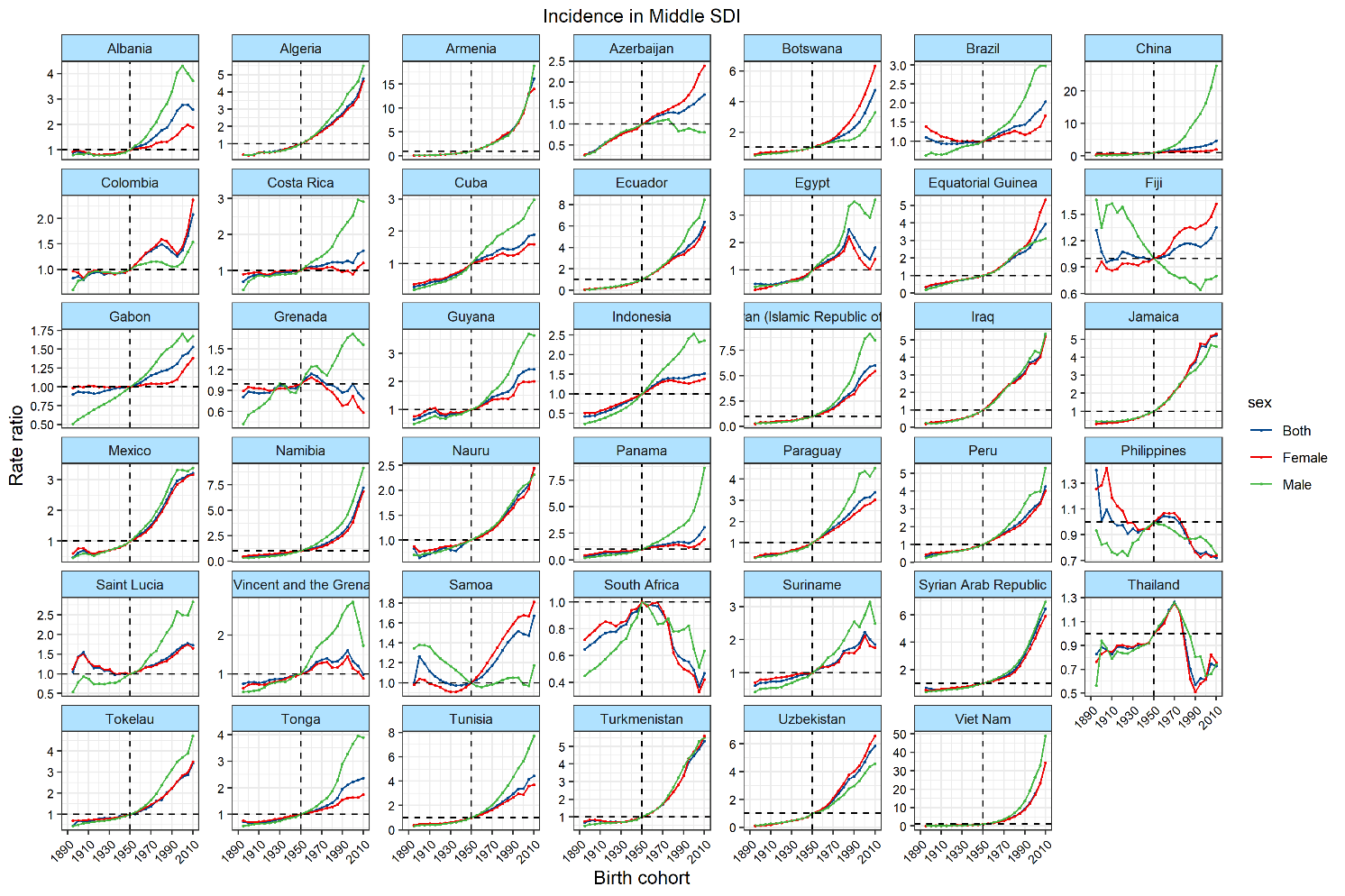


**Supplementary Figure 12. Incidence cohort effect in middle SDI countries.** Cohort effects on thyroid cancer incidence in middle-SDI countries. Cohort effects are shown by the relative risk of incidence and computed as the ratio of age-specific rates from the 1895 cohort to the 2010 cohort, with the referent cohort set at 1950.


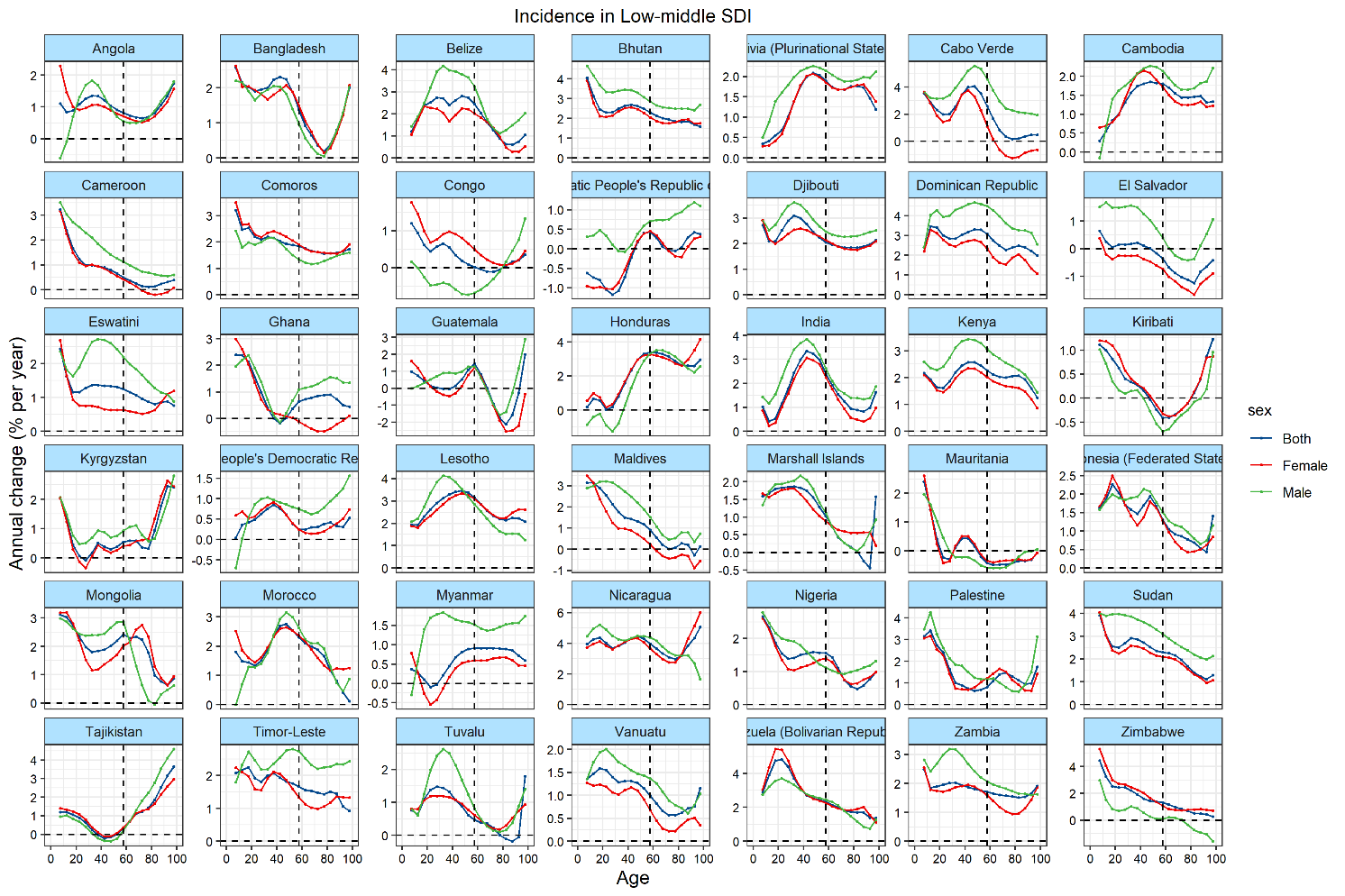


**Supplementary Figure 13. Incidence local drift in low-middle SDI countries.** The local drifts of thyroid cancer incidence in low-middle-SDI countries, 1990-2019. Local drifts of thyroid cancer incidence (estimates from age-period-cohort models) for 19 age groups (5−9 to 95 plus years), 1990−2019.


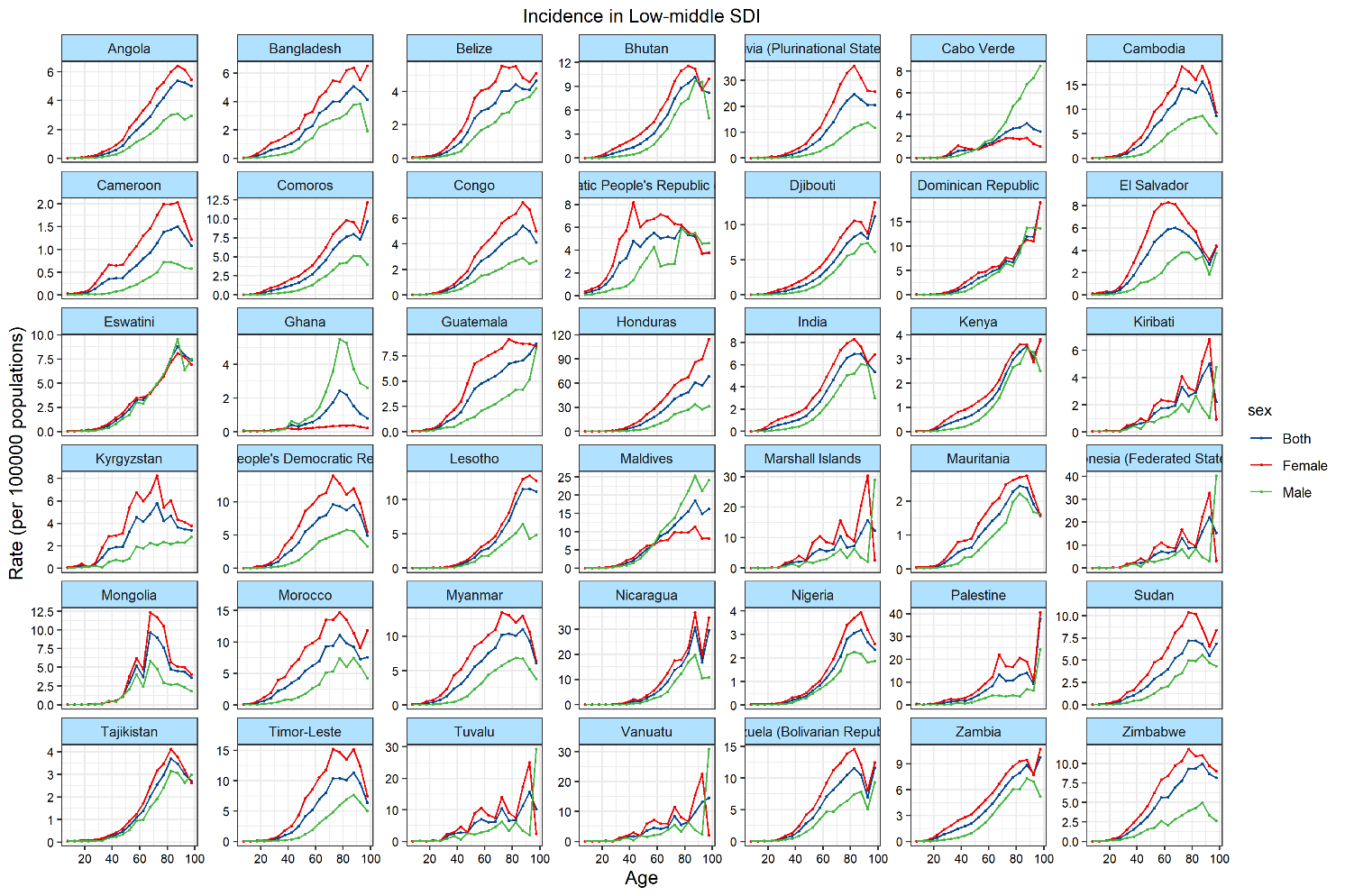


**Supplementary Figure 14. Incidence age effect in low-middle SDI countries.** Age effects on thyroid cancer incidence in low-middle-SDI countries. Age effects are shown by the fitted longitudinal age curves of incidence (per 100,000 person-years) adjusted for period deviations. (B) Period effects are shown by the relative risk of incidence (incidence rate ratio) and computed as the ratio of age-specific rates from 1990−1994 to 2015−2019 (2000−2005 as the referent period).


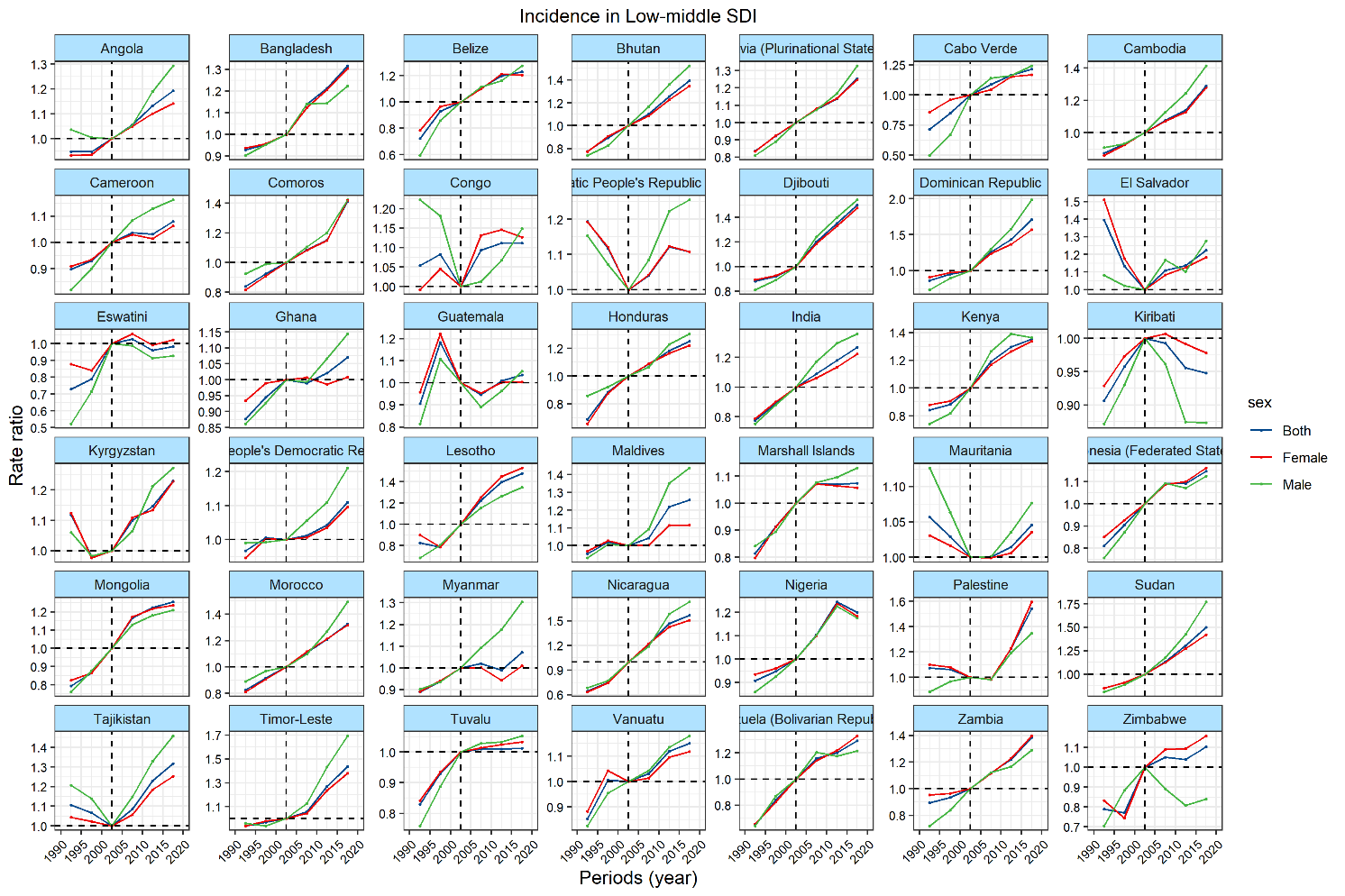


**Supplementary Figure 15. Incidence period effect in low-middle SDI countries.** Period effects on thyroid cancer incidence in low-middle-SDI countries. Period effects are shown by the relative risk of incidence (incidence rate ratio) and computed as the ratio of age-specific rates from 1990−1994 to 2015−2019 (2000−2005 as the referent period).


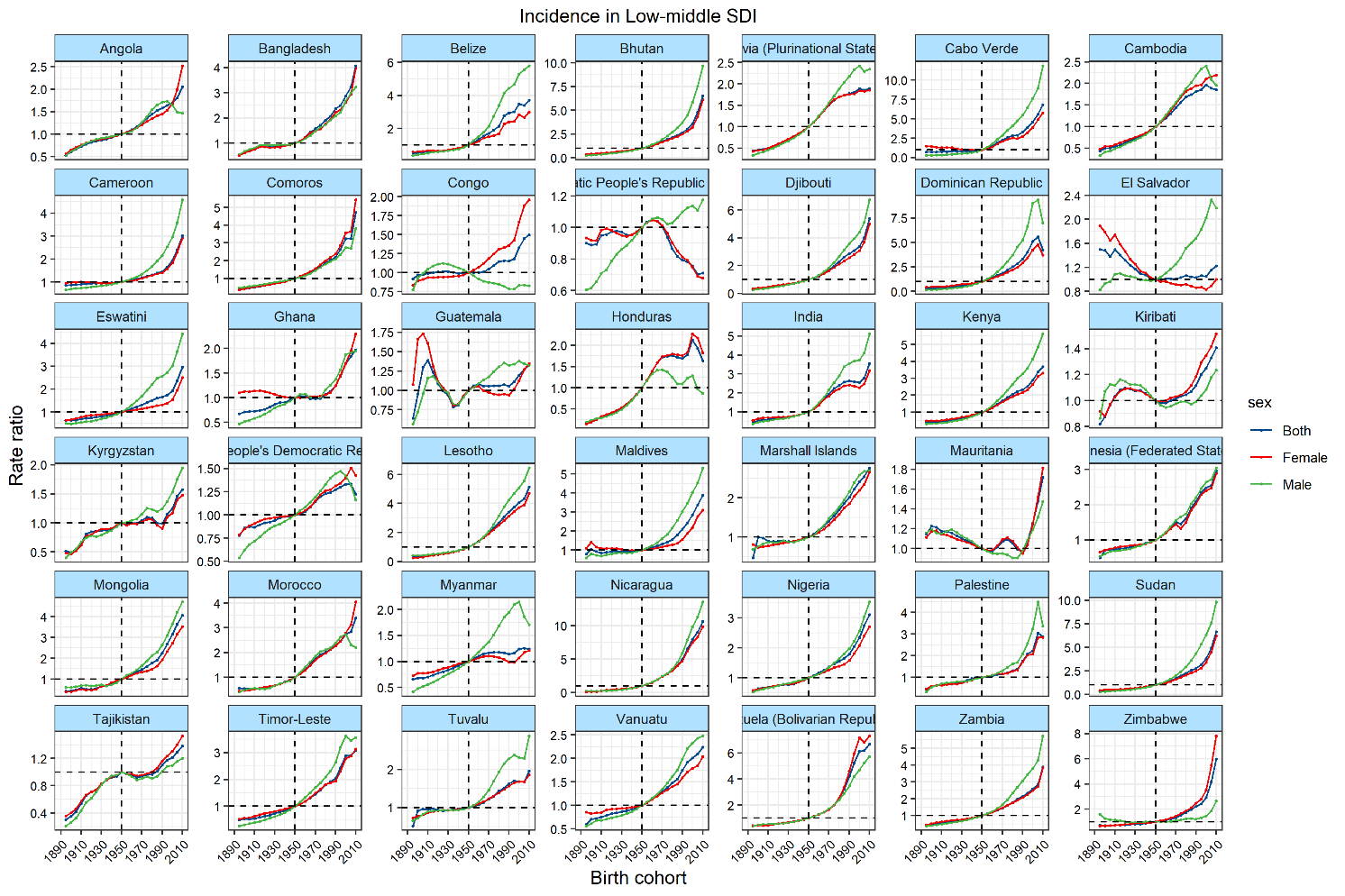


**Supplementary Figure 16. Incidence cohort effect in low-middle SDI countries.** Cohort effects on thyroid cancer incidence in low-middle-SDI countries. Cohort effects are shown by the relative risk of incidence and computed as the ratio of age-specific rates from the 1895 cohort to the 2010 cohort, with the referent cohort set at 1950.


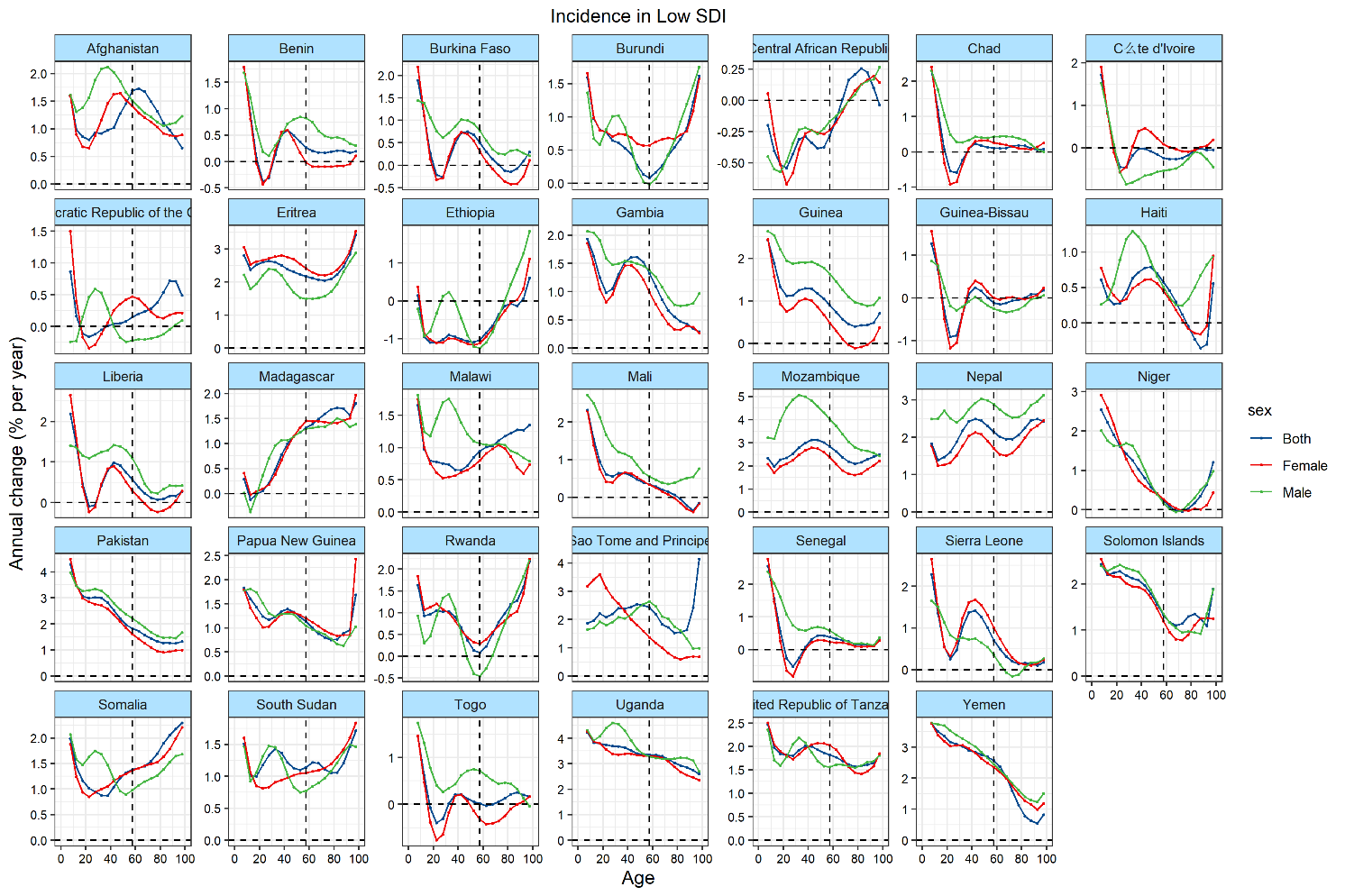


**Supplementary Figure 17. Incidence local drift in low SDI countries.** The local drifts of thyroid cancer incidence in low-SDI countries, 1990-2019. Local drifts of thyroid cancer incidence (estimates from age-period-cohort models) for 19 age groups (5−9 to 95 plus years), 1990−2019.


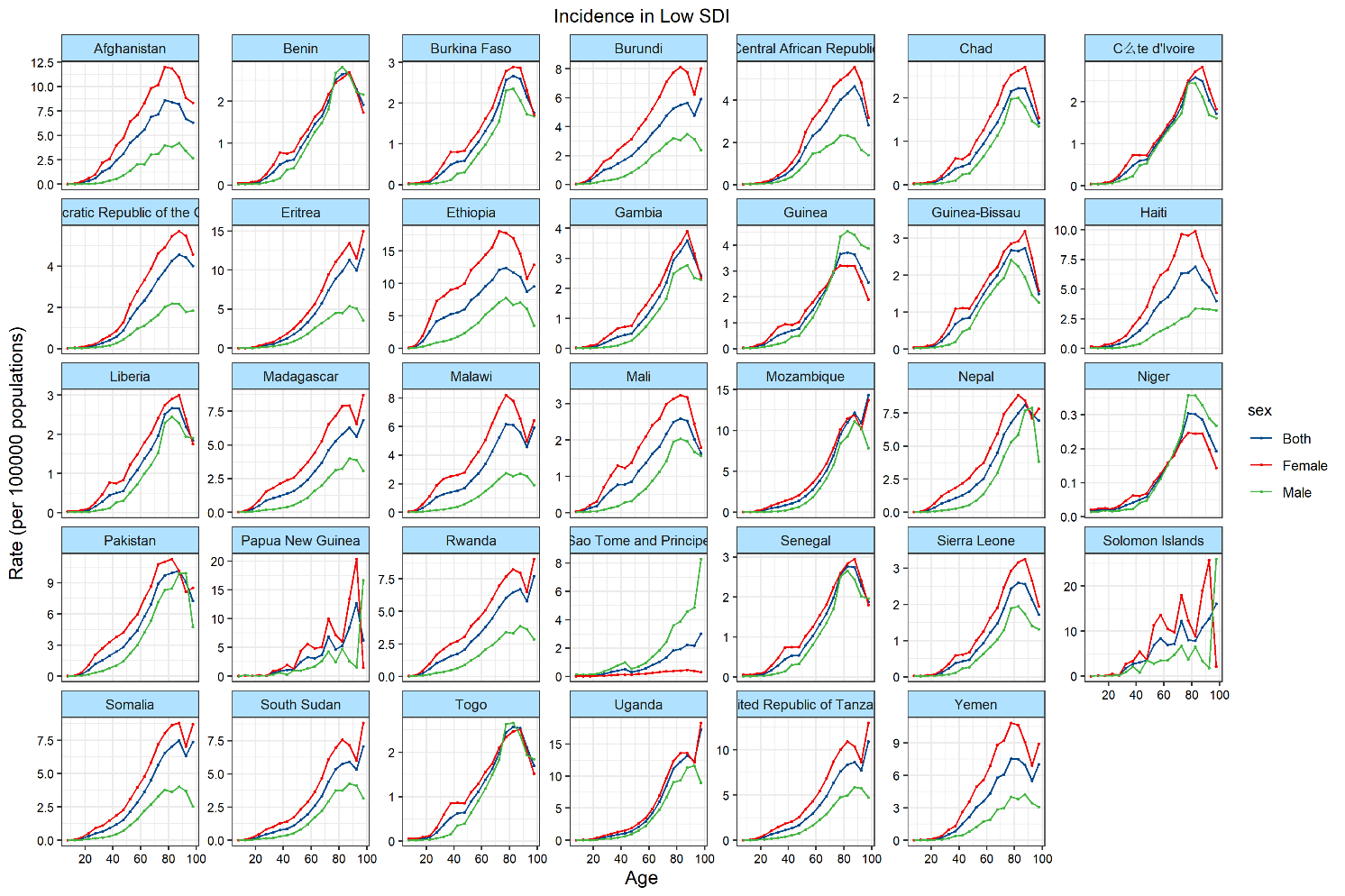


**Supplementary Figure 18. Incidence age effect in low SDI countries.** Age effects on thyroid cancer incidence in low-SDI countries. Age effects are shown by the fitted longitudinal age curves of incidence (per 100,000 person-years) adjusted for period deviations. (B) Period effects are shown by the relative risk of incidence (incidence rate ratio) and computed as the ratio of age-specific rates from 1990−1994 to 2015−2019 (2000−2005 as the referent period).


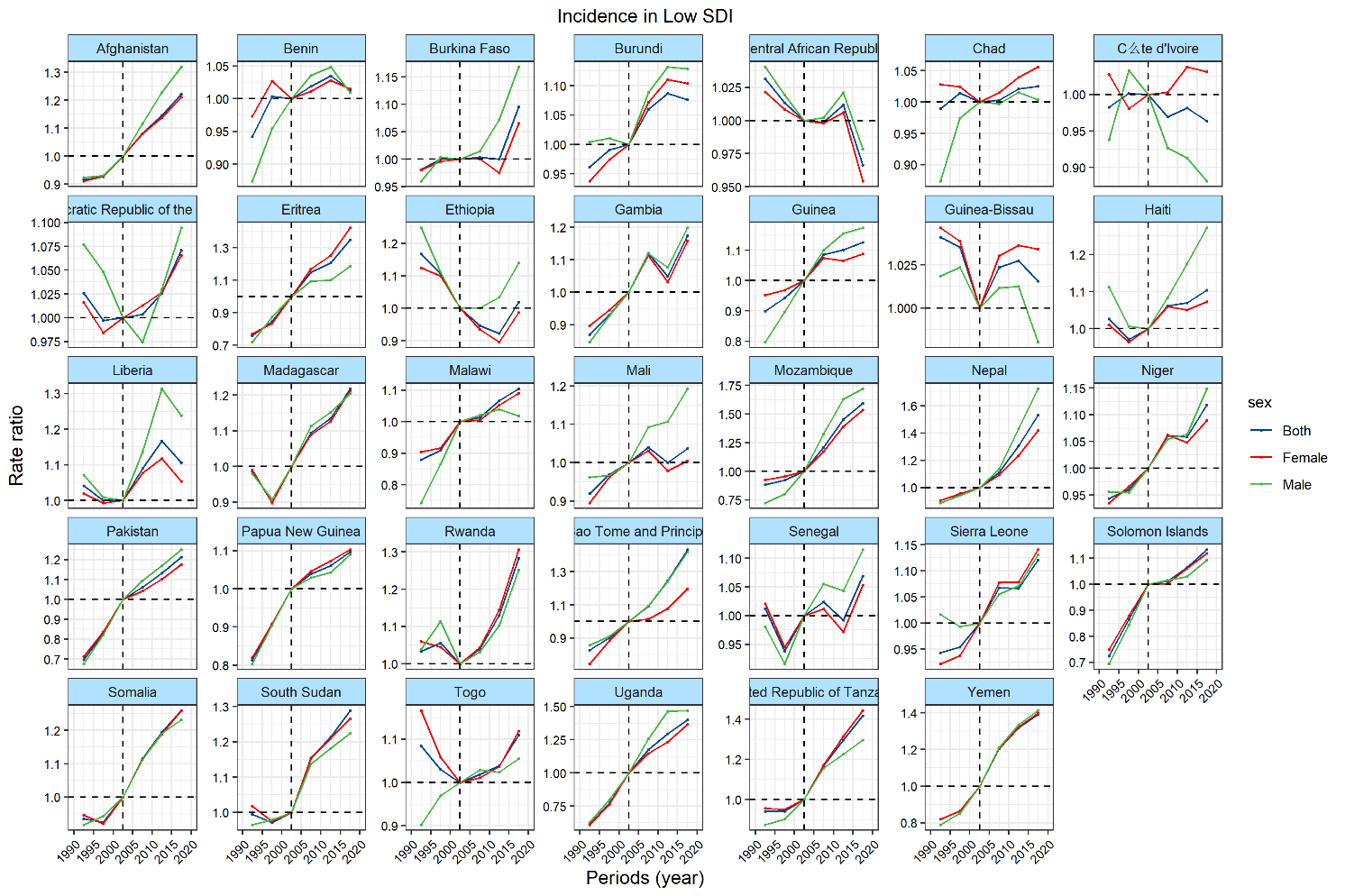


**Supplementary Figure 19. Incidence period effect in low SDI countries.** Period effects on thyroid cancer incidence in low-SDI countries. Period effects are shown by the relative risk of incidence (incidence rate ratio) and computed as the ratio of age-specific rates from 1990−1994 to 2015−2019 (2000−2005 as the referent period).


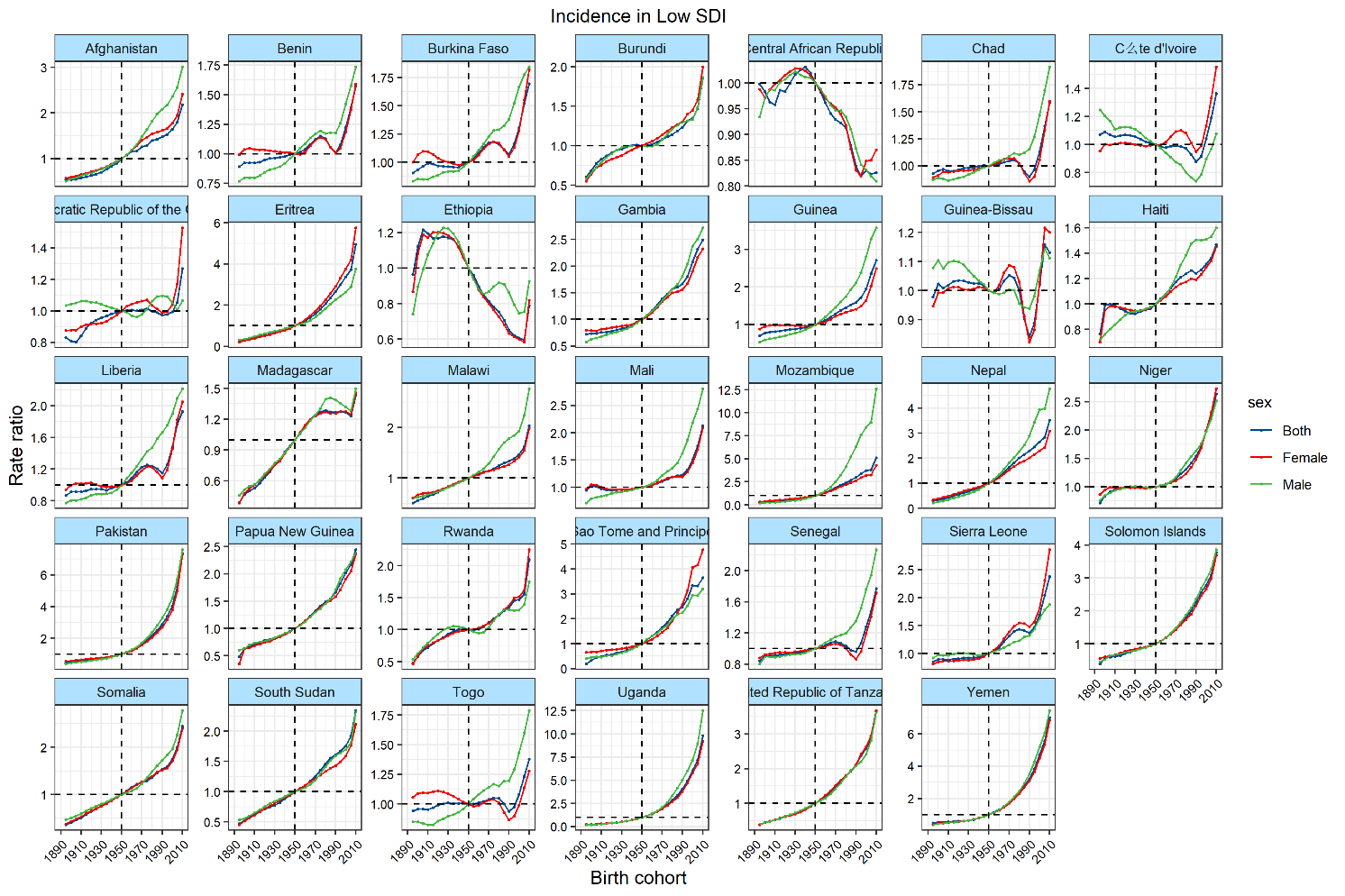


**Supplementary Figure 20. Incidence cohort effect in low SDI countries.** Cohort effects on thyroid cancer incidence in low-SDI countries. Cohort effects are shown by the relative risk of incidence and computed as the ratio of age-specific rates from the 1895 cohort to the 2010 cohort, with the referent cohort set at 1950.
